# GamblingLess: In-The-Moment: a mixed-methods acceptability and engagement evaluation of a gambling just-in-time adaptive intervention

**DOI:** 10.1186/s13722-025-00608-4

**Published:** 2025-10-14

**Authors:** N. A. Dowling, S. S. Merkouris, C. J. Greenwood, G. J. Youssef, A. C. Thomas, C. O. Hawker, D. I. Lubman, S. N. Rodda

**Affiliations:** 1 School of Psychology, Deakin University, Burwood, Australia; 2 Monash Addiction Research Centre and Eastern Health Clinical School, Faculty of Medicine, Nursing and Health Sciences, Monash University, Melbourne, Australia; 3 Turning Point, Eastern Health, Melbourne, Australia; 4 Department of Psychology and Neuroscience, Auckland University of Technology, Auckland, New Zealand

**Keywords:** Mobile health, MHealth, Just-in-time adaptive intervention, App, Ecological momentary intervention, Microrandomized trial, Gambling

## Abstract

**Background:**

Mobile health interventions, particularly dynamic Just-In-Time Adaptive Interventions (JITAIs), can overcome barriers to gambling treatment by offering timely, accessible support in people’s everyday lives. *GamblingLess: In-The-Moment* is a theoretically-informed and evidence-based app-delivered JITAI to people who want to quit or reduce their gambling. The JITAI aims to reduce gambling symptom severity through short-term reductions in the likelihood of gambling episodes by improving cognitive vulnerability (craving intensity, self-efficacy, or positive outcome expectancies). It administers three daily ecological momentary assessments (EMAs) to deliver tailored interventions in moments of cognitive vulnerability. Given that intervention acceptability and engagement are likely to improve clinical outcomes, this study aimed to comprehensively examine these constructs for *GamblingLess: In-The-Moment*.

**Methods:**

A 28-day micro-randomised trial (MRT) was conducted, with a supplementary six-month within-group follow-up evaluation and a mixed-methods acceptability/engagement evaluation. The acceptability/engagement evaluation included: (1) app use and engagement indices across the MRT (*n* = 192; 66% male; age_median_=35 years); (2) app acceptability measures administered at post-intervention (*n* = 161; 84% completion rate), and (3) semi-structured interviews (*n* = 11).

**Results:**

App use and engagement indices indicated that the JITAI was an attractive option for gambling support. Participants completed 5,116 EMAs (compliance rate = 32%, averaging 27 EMAs), spent an average of 30 min in the app, and completed an average of nine intervention activities from a pool of 53 activities they could repeatedly access. Subjective quality and perceived impact scores well exceeded minimally acceptable standards but 77% of participants preferred a hybrid push-pull approach and many endorsed less frequent EMAs (52%) but a longer program (58%). Participants also endorsed additional features, such as in-person support, motivational messages, gambling feedback, saving favourite activities, online discussion boards, virtual computer coaches, and in-app rewards. Interviews revealed two distinct themes: (1) facilitation of gambling reductions through check-ins/availability, personal tailoring, seamless and holistic support, and treatment experience suitability; and (2) promoting behaviour change through enhanced awareness, goal-setting, skill-building, and positive habit formation.

**Conclusions:**

*GamblingLess: In-The-Moment* was highly accepted and was generally perceived as effective in supporting reductions in gambling behaviour. The findings underscore the iterative process for JITAI development and highlight several avenues for its optimisation, particularly in relation to enhancing user engagement and reducing user fatigue.

**Trial registration:**

The evaluation was registered with the Australian New Zealand Clinical Trials Registry (ACTRN12622000490774) in March, 2022.

**Supplementary Information:**

The online version contains supplementary material available at 10.1186/s13722-025-00608-4.

## Introduction

Gambling disorder or problems are associated with a high burden of harm across multiple life domains, including finances, wellbeing, and relationships [1]. Despite this, treatment uptake is low, with only one in five people with problem gambling and 1 in 25 people with moderate-risk gambling seeking professional support, non-professional support, or self-help [2]. Many barriers to accessing gambling treatment have been identified, including stigma, denial, shame, a preference for self-management, goals of abstinence, and resource constraints related to location, time, cost, and clinician availability [3–7].

### mHealth interventions

Mobile health (mHealth) interventions overcome many of these barriers by offering high accessibility, convenience, anonymity, immediacy, cost-effectiveness, and ease of use [8–14]. With global smartphone penetration around 70% [15], mHealth provides unprecedented access to evidence-based therapeutic content [8, 11, 13], especially for underserved populations, such as those living in rural and regional areas [10–12]. These interventions can deliver intervention content on-demand (“pull”) or at times based on an intervention protocol (“push”) [9, 15].

Just-In-Time Adaptive Interventions (JITAIs) are a collection of “push” mHealth interventions that use mobile and wearable devices to monitor fluctuating internal states and social contexts (e.g., through ecological momentary assessments [EMAs]) to determine the type, amount, and timing of support needed [8, 10, 15–19]. JITAIs provide dynamic support in moments of need, in real-time and real-life, to prevent negative health outcomes and promote positive health outcomes [8, 9, 15–18]. They use pre-specified *decision rules* that operationalise *decision points* (points in time where the protocol is enacted), *tailoring variables* (information collected to determine whether support is needed and user is receptive to support, e.g., via EMAs), and *intervention options* (differing supports available) [16–18]. Intervention protocols are underpinned by short-term (proximal) and long-term (distal) outcomes. The ability of JITAIs to provide tailored support with minimal user input makes them promising for extending the reach of evidence-based treatments among gambling populations.

### mHealth gambling interventions

Gambling mHealth interventions are emerging, with around 14 free publicly available gambling support apps available in app stores [20]. Many are “pull” interventions offering progress tracking, psychoeducation, and gambling assessments. Despite relatively high download rates, few are evidence-based, use established therapeutic models, or feature multiple behaviour change functions, expert involvement, or high-quality interactive content [20–22]. Moreover, few have been empirically evaluated. The only “pull” intervention with published evaluation data is *GAMBOT*, a ‘chatbot’ delivering monitoring, feedback, and therapeutic content [23, 24], the efficacy of which has been evaluated in two randomised controlled trials (RCTs). Ongoing evaluations of other “pull” interventions include an RCT for *Manaaki*, which is a culturally-responsive app for New Zealand [25, 26]; and a single-arm evaluation of *Reset* under real-world conditions, which is an app offering cognitive-behaviour therapy (CBT) and motivational interviewing in Victoria, Australia [27]. There is also a limited evaluation (focus groups and interviews) of the JITAI, *sPGeTTI*, that provides geolocation sensing, self-monitoring, psychoeducation, and help links in New Zealand [28].

The only JITAIs with comprehensive evaluation data are three apps in Australia using EMAs to monitor internal states and social contexts: *Gambling Habit Hacker*, *GamblingLess: Curb Your Urge*, and *GamblingLess: In-The-Moment*. *Gambling Habit Hacker* aims to enhance adherence to gambling expenditure limits by providing tailored behaviour change techniques related to goal setting, action planning, coping planning and self-monitoring in response to low intention strength, low goal self-efficacy, low urge self-efficacy, and the presence of high-risk situations reported in EMAs [29–31]. Derived from an evidence-based online self-directed gambling program (*GamblingLess)* [32], *GamblingLess: Curb Your Urge* delivers tailored craving management activities in response to high craving intensity reported in EMAs [33, 34]. Finally, *GamblingLess: In-The-Moment* built on *GamblingLess: Curb Your Urge* to deliver tailored intervention options targeting not only craving intensity, but also other cognitive processes underpinning relapse (self-efficacy and positive outcome expectancies) in response to high craving intensity, low self-efficacy, and high positive outcome expectancies reported in EMAs [29, 35, 36]. *GamblingLess: Curb Your Urge* was evaluated using a single-arm pilot study. In contrast, *Gambling Habit Hacker* and *GamblingLess: In-The-Moment* were developed as part of the same project and were subject to both optimisation (microrandomised trials [MRTs], which are a form of sequential factorial design in which every participant serves as their own control [8, 16]) and evaluation trials (using six-month within-group follow-up evaluations). Collectively, these evaluations demonstrate improvements in gambling symptom severity, gambling frequency and expenditure, cravings, self-efficacy, positive outcome expectancies, psychological distress, and wellbeing [23, 24, 33, 36, 37].

### Acceptability and engagement of mHealth gambling interventions

The formative state of mHealth gambling intervention development and evaluation highlights the need to examine app acceptability and engagement prior to implementation and dissemination [34, 38–40]. There is no consensus definition of acceptability, which has been defined as affective attitudes, usage intentions, actual usage, and satisfaction [38, 39, 41]. A contemporary definition, which suggests that acceptability is a multidimensional construct reflecting perceived appropriateness based on cognitive and emotional responses to the intervention, has been operationalised by the Theoretical Framework of Acceptability (TFA), which comprises seven component constructs: affective attitude, burden, perceived effectiveness, ethicality, intervention coherence, opportunity costs, and self-efficacy [38]. In addition to incorporating the component constructs of acceptability (cognitive and emotional responses), this operational definition of acceptability provides a hypothesis that cognitive and emotional responses are likely to influence behavioural engagement with the intervention [38], which is most commonly measured using system usage data [42]. There is also no consensus definition of engagement, but it has been defined as a multidimensional construct relating to behavioural (e.g., frequency, amount, depth, and duration of intervention use), as well as cognitive and affective facets (e.g., attention, interest, and enjoyment [39]). There has been a differentiation between micro- and macro-level engagement, whereby micro-level engagement refers to the immediate, moment-to-moment interaction with intervention in terms of both intervention use (e.g., number of activities completed) and the quality of user experience (e.g., user interest and attention), while macro-level engagement captures the broader involvement in the behaviour change process (e.g., motivation to change behaviour), which is linked to the behavioural goals of the intervention (e.g., how the intervention may have helped the user to achieve behavioural goals) [43]. It has been argued that acceptability can be viewed as an emergent characteristic of a complex, adaptive system composed of interacting elements (e.g., beliefs, knowledge, affective attitude), which influences, and is influenced by, user engagement and intervention effectiveness [39]. Research therefore emphasises the importance of examining intervention acceptability and engagement as factors that can influence intervention effectiveness, thereby enabling intervention optimisation [39].

Recent evidence demonstrates the general acceptability of mHealth gambling interventions, with high confidence in their effectiveness and support for their anonymity, as well as neutral levels of scepticism or perceived deficits [44]. However, the average Mobile App Rating Scale (MARS [45]) scores for subjective quality (2.10) and perceived impact (2.63) of the 14 publicly available gambling support apps [20] fail to meet the minimum acceptability score of 3 (out of 5) [46]. Few of these apps include cognitive-behaviour therapy (CBT) content (29%), but those that do are rated higher in quality than those that do not [20], aligning with the broader evidence supporting CBT as the most effective psychological intervention for gambling [47, 48].

In contrast, the abovementioned gambling mHealth interventions that have been subject to empirical evaluation demonstrate promising acceptability and engagement using app use and engagement indices, app acceptability measures, or qualitative methods. App use and engagement indices reveal *GAMBOT* had 77% participant retention and use 23 out of 27 days [23]. *GamblingLess: Curb Your Urge* had a 47% EMA compliance rate and a 15% intervention compliance rate, with participants using it a median of 7 times [33]. *Gambling Habit Hacker* had a 30% EMA compliance rate, with participants spending approximately 34 min using the app over the 28-day trial, and weekly engagement decreasing to 48.3% in Week 4 of the trial [31]. Using app acceptability measures, experts and participants rated the EMA-based JITAIs (*GamblingLess: Curb Your Urge; GamblingLess: In-The-Moment; Gambling Habit Hacker)* as helpful, relevant, and easy to use, with subjective quality and perceived impact scores exceeding acceptable standards on the MARS [30, 33–35]. However, only 41.4% of participants indicated they would recommend *GAMBOT* to others [23]. Finally, qualitative feedback on the intervention content, usability, look and feel, engagement, and interactivity of these JITAIs is generally positive [28, 30, 33–35].

### Study aims

**Fig. 1:**
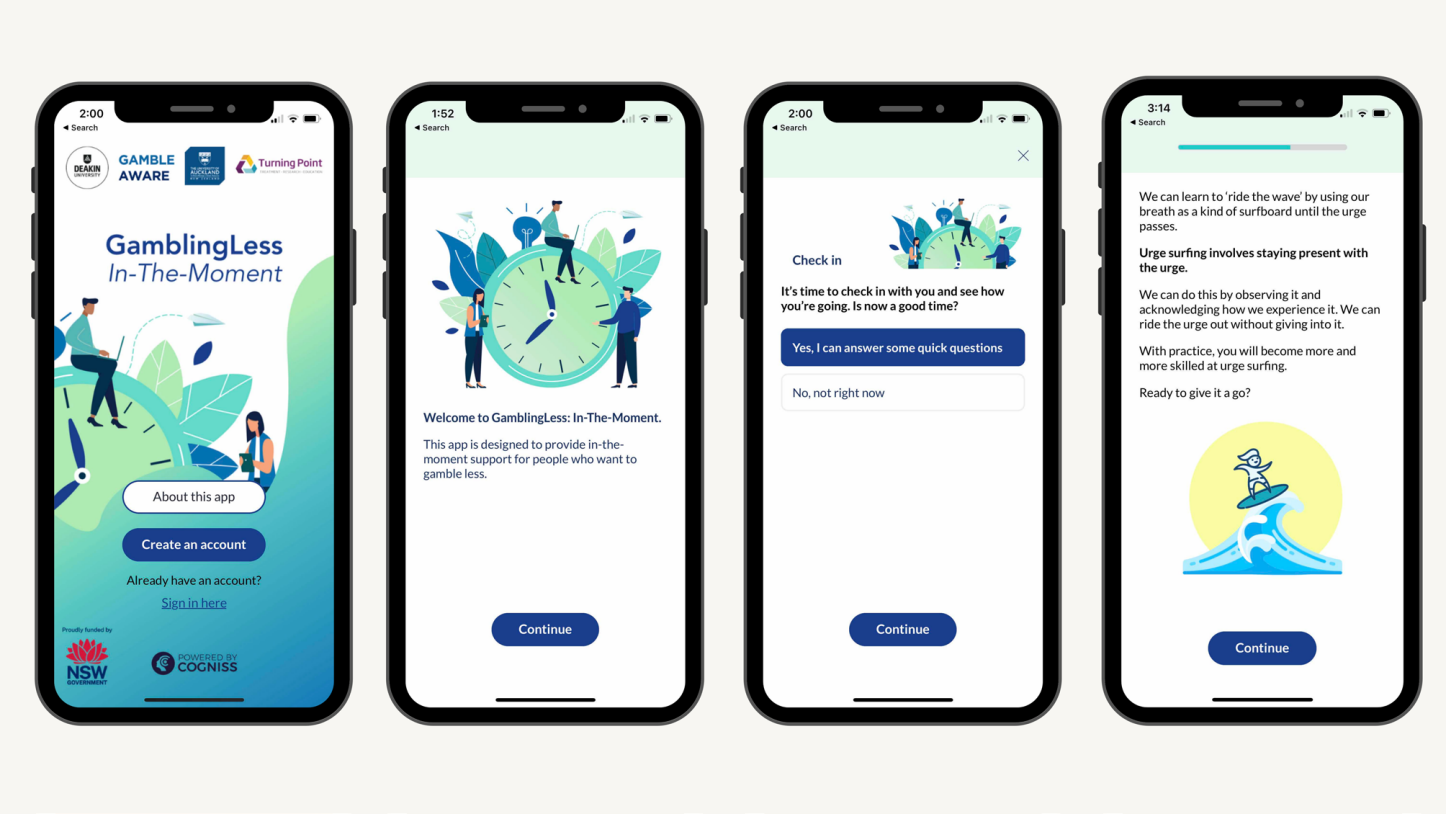
Illustrative screenshots of *GamblingLess: In-The-Moment*

Despite acceptability and engagement being necessary conditions for intervention effectiveness [38, 39] there are not yet any purposive in-depth acceptability or engagement evaluations of the small number of available gambling JITAIs. This study therefore aims to conduct a comprehensive evaluation of the acceptability of, and engagement in, of *GamblingLess: In-The-Moment*, which was embedded within a broader optimisation and preliminary effectiveness evaluation (28-day MRT and six-month within-group follow-up evaluation findings reported elsewhere [36]). Designed to support people who want to quit or reduce their gambling, this JITAI is a multi-faceted JITAI targeting gambling cravings, self-efficacy, and positive outcome expectancies (see Fig. 1), which provides a standalone or adjunctive treatment during active gambling periods or to prevent relapse. It is based on the Dynamic Model of Relapse, which theorises that temporary fluctuations in cognitive processes, such as cravings, self-efficacy, and positive outcome expectancies, act as phasic (transient) precipitating factors that interact with more tonic (stable) factors or other phasic factors to affect the likelihood of lapse or relapse [49]. It also draws on cross-sectional empirical evidence linking these cognitive processes to gambling behaviour, symptom severity, and relapse [35], as well as emerging EMA studies finding that these cognitive processes act as phasic precipitants of gambling behaviour and/or interact with tonic or other phasic factors to predict future gambling behaviour [50–52]. The intervention therefore aims to reduce gambling symptom severity in the long-term (distal outcome) and the likelihood of gambling episodes in the short-term (primary proximal outcome) by improving craving intensity, self-efficacy, and positive outcome expectancies in the moment (secondary proximal outcomes).

Operationalised using a contemporary JITAI design framework [16–18], this JITAI uses *decision rules* to determine the provision of an *intervention option* targeting one of these cognitive processes (*Curbing Cravings*, *Tackling Triggers*, and *Exploring Expectancies* modules) to users, based on their state of cognitive vulnerability (high craving intensity, low self-efficacy, or high positive outcome expectancies: *tailoring variables*) identified through three-times daily EMAs over the 28-day trial period (*decision points*) (see Procedure section for more detailed information about these decision rules; and [29] for a rationale for the selection of these decision rules). It therefore tailors the type of support based on responses on the tailoring variables, the timing of support via multiple EMAs per day, and the amount of support using an intervention loop, in which users are offered additional intervention activities from the same intervention module if needed.

Preliminary usability testing with 13 stakeholders (gambling clinicians, researchers, end users) supported its acceptability in terms of engagement, functionality, aesthetics, information, quality, perceived impact, helpfulness, ease of use, and relevance [35]. The overall MARS app quality score was 3.57, with good subjective quality (score of 4.21) and perceived impact (score of 3.40). Users noted the app’s greatest impact was likely on knowledge, intention to change, and help-seeking, followed by awareness, attitudes, and behaviour change.

The 28-day MRT of *GamblingLess: In-The-Moment* [36] demonstrated some promising findings. There were no associations between the tailored intervention condition and the no-intervention control condition in any proximal outcome, likely due to reduced statistical power due to a low incidence of gambling episodes during the trial and/or an unexpected impact of the no-intervention condition. However, the tailored intervention condition reduced craving intensity and improved self-efficacy when EMAs were collapsed across each day. The *Tackling Triggers* and *Exploring Expectancies* intervention options also improved subsequent self-efficacy and positive outcome expectancies, respectively. Comparisons of participant responses on each EMA tailoring variable and the same EMA item administered after they had completed the first and last intervention activity in the intervention loop revealed medium to very large improvements in all secondary proximal outcomes (craving intensity, self-efficacy, and positive outcome expectancies).

The supplementary within-group follow-up evaluation (i.e., no control group) at pre-intervention, post-intervention, and six-month follow-up demonstrated small-medium to very large improvements in all distal outcomes (gambling symptom severity, gambling frequency, expenditure, craving severity, self-efficacy, and positive outcome expectancies). At six-months, recovery or improvement in gambling symptom severity was reported by 66.46% of participants. Subjective quality and perceived impact scores, but not the number of EMAs completed, number of interventions completed, number of activities completed, or week of intervention, were associated with recovery or improvement in gambling symptom severity, suggesting a need to further examine user acceptability and engagement of the intervention.

## Methods

### Study design

This study involved a mixed-methods acceptability and engagement evaluation of *GamblingLess: In-The-Moment*, embedded within a broader evaluation that included the 28-day MRT and six-month within-group outcome evaluation (findings reported elsewhere [36]). This evaluation was designed to evaluate the acceptability of the app itself, rather than the trial conditions. It used data collected during the MRT and six-month follow-up period but was analysed at the study’s conclusion. Three methods were employed: (1) app use and engagement indices (across the MRT and six-month follow-up periods); (2) app acceptability measures (administered in the post-intervention survey), and (3) semi-structured interviews (conducted between 1 and 3 months after the MRT period). Evaluation findings are presented for each method and interpreted collectively in the Discussion section. Reporting is compliant with the Mixed Methods Appraisal Tool [53] and the Consolidated Criteria for Reporting Qualitative Research (COREQ [54]). During the 28-day MRT, participants accessed the intervention on a ‘push’ basis, with the micro-randomisation protocol determining when and what intervention to deliver. From post-intervention to the six-month follow-up evaluation, participants accessed tailored intervention content on an on-demand ‘pull’ basis through participant-initiated EMAs. During this time, no notifications were delivered and no micro-randomisation occurred. The MRT was registered with the Australian New Zealand Clinical Trials Registry (ACTRN12622000490774) and the protocol was published [35]. Full details relating to the development and evaluation of the JITAI are available elsewhere [29, 35, 36]. All evaluation components were approved by the Deakin University Human Research Ethics Committee. 

### Participants

Participants for the MRT were recruited in Australia from March 28 to December 14, 2022, via convenience methods, including website, social media, and online forum advertising. Eligibility criteria included: (1) current Australian residence; (2) being at least 18 years of age; (3) a willingness to install the *GamblingLess: In-The-Moment app* on their internet-enabled smartphone; (4) a willingness to receive app notifications; (5) English language fluency; and (6) wanting support for their own gambling. The app platform is continuously updated to meet Apple and Google standards, ensuring compatibility with the latest smartphones and software. While the company officially supports the last two versions of Android and iOS, GamblingLess: In-The-Moment also functions on some older versions. The intervention in this pragmatic trial was available to any interested person who gambled, regardless of gambling symptom severity, treatment goal (abstinence or non-abstinence), or current treatment engagement.

The sample for the app use and engagement indices evaluation comprised the analytic sample for the MRT (*n* = 192; age_median_=35 years, range: 18–72). As indicated in Additional file 1, most participants were male (66.15%), of Australian descent (75.52%), resided in Australia’s two most populous states (New South Wales and Victoria) (75.52%), and earned between AUD$25,000 and AUD$99,999 (65.50%). With a mean Index of Relative Socio-Economic Advantage and Disadvantage (IRSAD [55]) of 1011.1 (SD = 75.9), the sample had a slightly higher socioeconomic status than the national average (standardised mean of 1000). Problematic gambling was most common on electronic gaming machines (EGMs: 65.63%), racing (53.65%), and sports or events (47.40%). Their average gambling symptom severity score on the Gambling Symptom Assessment Scale (G-SAS [56]) was classified in the severe category and their past-month gambling frequency and expenditure was 8 and over AUD$3,300, respectively. Of the 192 participants, 161 (83.85%) completed the post-intervention survey, including the app acceptability measures.

Participants for the semi-structured interviews were sampled to broadly reflect the wider pool of trial participants in terms of gender and app use, with a focus on NSW residents at the funding body’s request. As indicated in Additional file 2, the final sample included 11 participants (eight men, three women), aged from 23 to 74 years. This met the goal of at least 10 participants matching the gender ratio after the first round of recruitment. Approximately half of the interviewees resided in NSW. To diversify insights, participants were selected based on app use (EMAs completed) tertiles after the first round of recruitment: high (59 + EMAs), moderate (9–58 EMAs), and low (4–8 EMAs). Participants with fewer than four completed EMAs were excluded due to insufficient app use for meaningful feedback.

### Measures

#### App use and engagement indices

App use and engagement indices were collected across the 28-day MRT to assess participant registration, app use (EMA compliance, app use duration, timing of app use, and intervention use), and specific app feature use (e.g., use of the intervention loop and *Get More Support* quick link). These indices were also assessed in the six-month post-MRT period.

#### Measures of app acceptability

The post-intervention survey assessed subjective quality, perceived impact, perceived helpfulness of additional app features, preferred app approach, and JITAI protocol preferences to inform the optimisation of the app. Subjective quality and perceived impact were evaluated using the Subjective Quality and Perceived Impact subscales of the MARS [45]. The 4-item Subjective Quality subscale measures app recommendation, future use, willingness to pay, and star rating, with each item rated on varying 3-5-point scales. The 6-item Perceived Impact subscale assesses the perceived influence on participant awareness, knowledge, attitudes, intention to change, help-seeking, and behaviour change, with each item rated on a 5-point scale from (1) *Strongly disagree* to (5) *Strongly agree*. The MARS is a widely used and validated measure of app quality [57], for which subscale scores of 3 or more indicate minimum acceptability [46].

The post-intervention survey also assessed the perceived helpfulness of proposed app improvements, including the involvement of a therapist, an e-coach, or peer support and their method of communication (SMS, chat, email, telephone, video-conferencing, and in person [face-to-face]), as well as additional features (online discussion board, on-demand access, motivational messages, virtual computer coach, gambling behaviour feedback, in-app rewards, and saving favourite activities). Each item was rated on a 5-point scale from (1) *Not at all helpful* to (5) *Very helpful*. The preferred app approach was assessed using response options of (1) *Push only*, (2) *Pull only*, and (3) *Both push and pull*. JITAI protocol preferences were evaluated in terms of how many check-ins participants preferred rated on a 5-point scale from (1) *1* to (5) *5 or more*, and the desired program length was rated on a 7-point scale from (1) *1 week* to (7) *more than 6 months*. A subsequent open-ended item collected any other feedback or suggested improvements.

#### Semi-structured interviews

In the pre-intervention survey, participants could opt in for a semi-structured interview to explore the app’s acceptability and users’ perceptions of its helpfulness in supporting them to reduce their gambling. A broad list of questions was employed to assess the acceptability of, and engagement in, the JITAI (but not the trial conditions): first experiences of using the app (e.g., what the participant was searching for, the check-in process); the experience of intervention activities and specific app features; additional features they would like to see included in the app; personal changes having used the app; and overall impressions of the app (e.g., the look and feel of the app). The semi-structured nature of the interviews enabled follow-up questions to probe topics of interest where these arose [58]. Pilot testing of the semi-structured interview schedule (Additional file 3) was conducted by members of the research team and the schedule was refined to gain deeper insights. The planned interview duration was between 30 and 45 min.

### Procedure

Participants downloaded *GamblingLess: In-The-Moment* for free from the Apple App Store or Google Play Store free of charge, completed onboarding (consent, account creation, pre-intervention survey, first EMA), and received an automated welcome email. During the next 28 days, they participated in the MRT, receiving semi-random app notifications three times daily: 8:30 − 11:00am, 1:00-3.30pm, and 5:30-8.00pm (decision points). Although existing theoretical and empirical evidence offers limited insight into the temporal relationships among proximal outcomes, these decision points were chosen to balance the risk of obscuring important temporal patterns with considerations of participant availability and receptivity, as well as assessment burden, cognitive overload, potential reactance, and premature dropout [16–18] (see [59] for more detailed information on the rationale for these decision points). Participants who did not complete onboarding or an EMA within 48 h of onboarding received a reminder email, followed by another reminder via phone, SMS, or email if they still failed to respond after another 48 h. No further contact was made if they remained unresponsive.

Participants who were unable to complete an EMA could use the *snooze* function. Participants had up to two hours to complete each EMA via the app or an auto-launch notification. Each EMA included 10 multiple choice items, including three items measuring craving intensity, self-efficacy, and positive outcome expectancies (tailoring variables, secondary proximal outcomes) and an event record, in which gambling episodes (primary proximal outcome) and associated expenditure were recorded. At each EMA, participants who scored 0 out of 4 on all three tailoring variables were not eligible for an intervention and received only an encouraging message. In contrast, participants who scored more than 0 out of 4 on one or more of the three tailoring variables were eligible for a tailored intervention (intervention options). With a maximum of 84 EMAs across the MRT, participants could be eligible to receive an intervention option 84 times.

In the MRT, participants who were eligible for an intervention at each EMA were micro-randomised to a tailored intervention condition or a no-intervention control condition on a 3:1 ratio (i.e., 75% chance of receiving the tailored intervention and 25% chance of receiving the no-intervention control). Participants eligible for multiple interventions had an equal chance of receiving each option (e.g., 37.5% chance for two options). Those micro-randomised to the no intervention control condition received a brief encouraging message to address their cravings, low self-efficacy, or positive outcome expectancies but no intervention content, after which their interaction with the app ended.

Participants micro-randomised to the tailored intervention condition received one of three intervention modules targeting cognitive vulnerabilities (detailed elsewhere [35]): (1) *Curbing Cravings* (10 craving management activities, such as distraction and urge surfing); (2) *Tackling Triggers* (25 activities enhancing self-efficacy across five high-risk situations: financial pressure, unpleasant emotions, social pressure to gamble, testing control, and conflict with others); and (3) *Exploring Expectancies* (18 activities addressing positive outcome expectancies: excitement, escape, money). They were presented with a dashboard presenting a list of activities, most of which are interactive and gamified, featuring audio, video, personalised feedback, quizzes, or embedded questions. On each intervention dashboard, there was also a *Pick For Me* quick link, which randomly delivered activities and each activity was preceded by a *Did You Know?* psychoeducational message relating to gambling cravings, self-efficacy, or positive outcome expectancies.

After completing an intervention activity, participants were prompted to re-answer the same EMA item (e.g., the craving intensity item after a *Curbing Craving* activity) and receive feedback on their progress. If their post-intervention score was 0, they received a brief encouraging message and their interaction with app ended. If their score remained above 0, they returned to the relevant dashboard to choose another activity. This intervention loop continued their score dropped to 0 or they closed the app. Participants were informed that they could close the app at any time to stop the loop. The app also included a *Get More Support* feature on each dashboard linking to Australian gambling help services.

Participants completed the post-intervention survey and a six-month follow-up survey via the Qualtrics platform. An email was sent a week before the six-month follow-up survey was due. Up to three follow-up reminders (email, phone, SMS) were sent over the next three weeks. Participants who did not complete the post-intervention survey were still invited to complete the six-month follow-up survey.

Participants for the semi-structured interviews were selected from eligible trial participants who consented in the pre-intervention survey and completed the post-intervention survey. Recruitment occurred in five rounds across the trial, with email invitations followed by and email reminder a week later. Interviews (23 to 46 min duration) were conducted by an experienced female gambling researcher with a PhD qualification and extensive experience in conducting semi-structured interviews (AT). Prior to each interview, this researcher had minimal contact with the interview participants, with the exception of email contact to arrange interview times. No other non-participants were present during each interview. The interviews were conducted via telephone or video-conferencing (audio-only) and recorded using the Audacity platform (for telephone interviews) and Zoom recording (for video-conferencing interviews). De-identified interviews were transcribed verbatim using a professional transcription service (for telephone interviews) and Zoom automatic transcription (for video-conferencing interviews), which were manually checked by members of the research team (RR, AT). The first interview transcript was reviewed by other members of the research team (ND, SR) to ensure that requisite data were adequately collected. None of the interview participants accepted the offer of the opportunity to review their transcripts.

The reimbursement schedule ($AUD via e-gift vouchers) included: $1 per completed EMA with a $20 bonus for completing over 75% of the EMAs (to a maximum of $100); $50 per completed post-intervention and follow-up survey; and $30 for completion of the interview.

### Statistical analysis

Quantitative data on app use and engagement indices and app acceptability measures were analysed in Stata 17 using descriptive statistics (e.g., means, standard deviations, medians, interquartile ranges, frequency counts, and proportions). Semi-structured interview data were analysed using reflexive thematic analysis [60, 61]. Within the constraints of the aim, an inductive approach, in which the data drove identification of the final themes, was employed [60]. In Excel, a female research assistant experienced in qualitative data analysis (RR) conducted the initial analysis by reading and re-reading responses to enhance data familiarisation then coding small sections of the data according to meaning. These codes were then reviewed and compared to each other for similarities and differences and clustered at a higher level, generating a list of initial themes. This initial list of themes and associated data and codes was then reviewed and discussed with an experienced gambling researcher with extensive experience in thematic data analysis (AT), considering the relevance of the themes to the research aim, resulting in a jointly developed revised list of themes. This list was discussed with two other experienced gambling researchers (ND, SR), resulting in the final list [60]. Member checking was not employed, but a cross-check between the reported findings and annotations made during data analysis ensured that key points were maintained. Illustrative quotes were minimally used to highlight key themes, ensuring accurate interpretation and consistency between the findings and their interpretation. Quotes are presented verbatim, with only minor grammatical or expression corrections made to enhance their readability. To protect confidentiality, the names of all interview participants were replaced with a participant number, which was used in reporting. To increase transparency, individual quotes used in the presentation of results have been coded to reflect the participant gender, age, G-SAS gambling symptom severity category, and app use (high, moderate or low).

## Results

### App use and engagement indices

App use and engagement indices across the 28-day MRT period related to participant registration, app use, and the use of specific app features. Post-MRT app use and engagement indices were also examined.

#### Participant registration

The aim was to recruit 200 participants for the MRT, with a sample size of 120 participants providing greater than 80% power to detect a small true intervention effect size for the primary proximal outcome (relative risk = 1.21, α = 0.05; availability parameter = 0.3; randomization *P* =.25; outcome without intervention *P* =.25) [62]. Data from 329 individuals were recorded during the recruitment period. Of these, 27 were ineligible as they did not meet the eligibility criteria: seven signed up as ‘family or friends’, 18 signed up as ‘other stakeholders’, one was aged under 18 years, and one indicated non-Australian residence. The remaining 302 people met the eligibility criteria, which equates to an average of 36 eligible users recruited per month. Of these, 34 people were removed for other reasons (22 due to a technical malfunction, one due to multiple sign ups, 10 who signed up shortly after signing up to a concurrent app evaluation, and one with un-linkable data); 41 people failed to complete onboarding (35 who failed to complete the pre-treatment survey and 6 who failed to complete the EMA immediately after onboarding [Day 0: day of onboarding]); and 35 people were not included in the analytic sample as they only completed the EMA after onboarding (Day 0). The final analytic sample comprised 192 participants who completed at least one EMA during the 28-day MRT (Days 1–28). The median time to complete the pre-intervention survey during was approximately 10 min (median = 10.47, IQR = 7.60–15.52).

#### App use across the 28-day MRT

##### EMA compliance

Overall, 5,116 EMAs were completed (compliance rate = 31.72%) by the 192 participants in the analytic sample. Participants completed an average of 26.65 EMAs (SD = 28.02, median = 10, IQR = 3-53.5, range = 1–82) of a possible 84 EMAs, with 13.54% of participants completing only one EMA. On average, participants responded to the *Check in Here* quick link on 12.57 days (SD = 11.11, median = 10, IQR = 2–26, range-1-28) and used the *Snooze* function less than once (M = 0.55, SD = 2.03, median = 0, IQR = 0–1, range = 0–26). A considerable proportion of participants (40.63%) completed more than one EMA within the allowed 2-hour period at least once (M = 1.01, SD = 2.22, IQR = 0–1, range = 0–19).

##### App use duration

 The median time to complete each EMA was half a minute (median = 0.52, IQR = 0.35–0.75), with participants across both treatment and control conditions spending around two-thirds of a minute (median = 0.65, IQR = 0.42–1.27) in the app each session (i.e., completing the EMA and relevant intervention activities, if delivered). The median time participants spent in the app across the 28-day MRT (including EMAs and intervention activities) was just over half an hour (median = 34.25, IQR = 6.42-369.64).

##### Timing of app use

 Additional file 4 presents the number of times participants used the app across the days and weeks of the MRT period. On average, participants used the app between 8 and 9 times during the morning (5:00am-12:00pm), afternoon (12:00pm-5:00pm), and evening (5:00pm-5:00am). The average number of times participants used the app reduced across the MRT from nearly 9 times in Week 1 to 6 times in Weeks 2 to 4. The proportion of participants using the app dropped from 100% in Week 1 to 57.29% in Week 2, 49.48% in Week 3, and 45.83% in Week 4.

##### Intervention use

 Overall, 179 participants (93.23%) in the analytic sample completed at least one intervention. Of the remaining 13 participants, 2 were never micro-randomised because they did not reach the intervention eligibility threshold on the tailoring variables at any point during the trial and 11 were randomised but only ever to the no-intervention control condition. Overall, the *Curbing Cravings* intervention option was used at least once by 108 participants (56.25%, 17.60% of all EMAs), the *Tackling Triggers* intervention option was used at least once by 138 participants (71.88%, 28.92% of all EMAs), and the *Exploring Expectancies* intervention option was used at least once by 130 participants (67.71%, 27.49% of all EMAs).

The number of intervention activities completed (including repeated activities within the intervention loop and across sessions) by participants overall and who were randomised to each intervention option are presented in Additional file 5. Overall, participants completed an average of nine activities across the three intervention options, with 1503 activities completed in total. Of the 108 participants micro-randomised to *Curbing Cravings*, 82.41% completed at least one activity, with an average of four activities completed and three different activities completed. Of the 138 participants micro-randomised to *Tackling Triggers*, 93.48% completed at least one activity, with an average of five activities completed and four different activities completed. Of the 130 participants micro-randomised to *Exploring Expectancies*, 90.00% completed at least one activity, with an average of four activities completed and three different activities completed.

The most commonly selected activities are presented in Additional file 6. Within the *Curbing Cravings* intervention option, the most commonly selected activity was *Delay and Distract* (distraction activity*)*, followed by *Breathe Through It* (breathing relaxation exercise video), *Anti-Autopilot* (mindfulness meditation exercise video), *Fast-Forward* (imagery rescripting exercise), and *Ten Steps to Stay Safe* (ten steps summarising the other activities in the module and importing participant-entered responses, if available). Within the *Tackling Triggers* intervention option, *Money Check* (gambling calculator activity) was the most commonly employed activity for the *Financial Pressures* group, *Defusing Difficult Thoughts* (cognitive defusion activity) was the most common activity for the *Unpleasant Emotions* group, *The “No” Word* (cognitive restructuring activity targeting assertiveness beliefs) and *Just Say No* (assertiveness training activity) were the most common activities for the *Social Pressure* group, *Willpower Breakdown* (cognitive restructuring activity targeting the abstinence violation effect) was the most common activity for the *Testing Control* group, and *My Style* (communication style activity) was the most common activity for the *Conflict with Others* group. Within the *Exploring Expectancies* intervention option, *Feedback* (personalised normative feedback activity) and *Tense and Relax* (progressive muscle relaxation exercise video) were the most commonly selected activities for the *Excitement Expectancies* group, *Feedback* (personalised normative feedback activity) was the most common activity for the *Escape Expectancies* group, and *Money Check* (gambling calculator activity) and *Feedback* (personalised normative feedback activity) were the most common activities for the *Money Expectancies* group.

#### Use of specific app features

The intervention loop was used by 92.18% of the 179 participants who used at least one intervention. Specifically, the intervention loop was used by 82.41% of the 108 participants who used the *Curbing Cravings* intervention option, 93.48% of the 138 participants who used the *Tackling Triggers* intervention option, and 90.00% of the 130 participants who used the *Exploring Expectancies* intervention option. For each intervention option, participants used the intervention loop a median of 1–2 times and left the intervention loop a similar number of times before reaching a score of 0 (no endorsement of the tailoring variables), which represents ineligibility for continued intervention use (Additional file 7).

The *Curbing Cravings* intervention option, but not the other two intervention options, included an additional optional brief psychoeducational component about how urges work, which was optional included prior to the presentation of the intervention activity dashboard. Participants opted to the view this information approximately half the time they were micro-randomised to this intervention option (*n* = 162, 49.54%). The *Get More Support* quick link was used by nearly one-quarter of participants (*n* = 45, 23.44%); overall, this link was used 132 times.

#### Post-MRT app use

Of the 192 participants in the analytic sample, 161 (83.85%) completed the post-intervention survey and 158 (82.29%) completed the six-month follow-up survey. The median time to complete the post-treatment survey was 14.00 min (IQR = 7.95), while the median time to complete the six-month follow-up survey was 6.24 min (IQR = 4.17). During the six-month follow-up period in which no notifications were provided, 25 (13.02%) participants used the app. These participants completed a total of 127 EMAs (M = 5.08, SD = 9.85, median = 1, range = 1–43) across the first four months of the follow-up period and none thereafter: Month 1 (61.42%), Month 2 (17.32%), Month 3 (20.47%), and Month 4 (0.79%). There were no differences in age (*p* =.392), gender (*p* =.254), or pre-intervention G-SAS gambling symptom severity (*p* =.453) between those participants who did or did not use the app in the six-month follow-up period. However, those who used the app in the six-month follow-up period engaged in a greater number of intervention activities during the 28-day trial (i.e., 9.82 activities higher; *p* =.002).

### Measures of app acceptability

The subjective quality of the app, the perceived impact of the app, the perceived helpfulness of additional app features, the preferred app approach, and JITAI protocol preferences were collected in the post-intervention survey.

#### Subjective quality and perceived impact

The subjective quality and perceived impact of *GamblingLess: In-The-Moment* is displayed in Table 1. On the MARS Subjective Quality subscale, participants provided mean ratings over the minimum acceptability score (≥ 3) (M = 3.20, SD = 0.86, median = 3.25), with 69.57% of participants providing scores over the minimum acceptability score. All items evidenced ratings over the minimum acceptability score, with the exception of whether they would be prepared to pay for the app, whereby only one-third of participants indicated they would be prepared to do so (33.54%). Most participants indicated they would give the app an overall rating of 3 stars or more (87.58%), recommend the app to people who might benefit from it (78.88%), and use the app more in the next 12 months if it was relevant to them (75.78%).

On the MARS Perceived Impact subscale, participants provided mean ratings over the minimum acceptability score (M = 3.92, SD = 0.92, median = 4.17), with 87.58% of participants providing scores over the minimum acceptability score. All items evidenced ratings over the minimum acceptability score, with the highest proportion of participants indicating the app had encouraged them to seek further help to reduce their gambling behaviour (92.55%), increased their intentions or motivation to reduce their gambling (91.30%), and increased their knowledge or understanding of gambling problems (90.06%), followed by increasing their awareness of the importance of reducing their gambling (88.20%), changing their attitudes toward reducing their gambling (87.58%), and decreasing their gambling (83.23%).

**Table 1:** MARS subjective quality and perceived impact subscale and item scores

MARS subscale	n (%) acceptable	Mean (SD)
Subjective Quality subscale	112 (69.57%)	3.20 (0.86)
Recommend to others	127 (78.88%)	3.51 (1.21)
Next-year use	122 (75.78%)	3.50 (1.23)
Prepared to pay	54 (33.54%)	2.17 (1.12)
Overall star rating	141 (87.58%)	3.65 (1.00)
Perceived Impact subscale	141 (87.58%)	3.92 (0.92)
Awareness	142 (88.20%)	3.96 (1.13)
Knowledge	145 (90.06%)	3.99 (1.06)
Attitudes	141 (87.58%)	3.86 (1.10)
Intention to change	147 (91.30%)	4.02 (1.03)
Help-seeking	149 (92.55%)	4.01 (1.00)
Behaviour change	134 (83.23%)	3.67 (1.18)

n = 161 participants (post-treatment evaluation sample)

#### Perceived helpfulness of additional app features

Participants were asked how the app could be improved by rating the helpfulness of a range of additional features in the post-intervention survey (*n* = 161; Table 2). Most participants indicated that additional support would be helpful, including that provided by a person who has recovered from their own gambling issues (83.85%), a qualified therapist or counsellor (83.23%), or an e-coach who has been trained to support various parts of the program (81.99%). The highest proportions of participants indicated that this support would be most helpful if delivered via chat (84.47%), SMS (76.40%), or in-person (face-to-face) (72.05%).

Participants also rated the potential helpfulness of several additional app features. Almost all participants (92.55%) indicated that being able to access the program any time they chose would be helpful. Over three-quarters of participants also ranked the remaining additional features as helpful: additional motivational messages (88.82%), feedback about changes to their gambling (such as graphs; 86.34%), ability to save their favourite activities (84.47%), an online discussion board (83.85%), a virtual computer coach (81.99%), and in-app rewards (75.78%).

**Table 2:** Perceived helpfulness of additional app features

	N (%) somewhat, moderately or very helpful	Mean (SD)
Additional support		
Qualified therapist/counsellor	134 (83.23%)	3.70 (1.14)
Person with lived experience	135 (83.85%)	3.80 (1.20)
E-coach	132 (81.99%)	3.65 (1.18)
Modality of additional support		
SMS	123 (76.40%)	3.55 (1.32)
Chat	136 (84.47%)	3.80 (1.18)
Email	102 (63.35%)	3.01 (1.33)
Telephone	105 (65.22%)	3.19 (1.39)
Video-conferencing	116 (72.05%)	3.00 (1.41
In person (face-to-face)	116 (72.05%)	3.49 (1.44)
App features		
Online discussion board	135 (83.85%)	3.67 (1.19)
Access anytime	149 (92.55%)	4.23 (1.02)
Motivational messages	143 (88.82%)	3.98 (1.12)
Virtual computer coach	132 (81.99%)	3.57 (1.19)
Gambling behaviour feedback	139 (86.34%)	3.75 (1.18)
In-app rewards	122 (75.78%)	3.57 (1.38)
Saving favourite activities	136 (84.47%)	3.73 (1.19)

n = 161 (post-treatment evaluation sample)

#### Preferred app approach

The app operated as “push” only during the 28-day MRT, during which participants could check in within 2 h of the 3-times a day EMA push notifications, and “pull” only in the six-month follow-up period, whereby participants could check in at any time but they were not sent any EMA push notifications. However, participants overwhelmingly indicated a preference for a hybrid design that combines both pull and push features (*n* = 124, 77.02%), with much smaller proportions of participants indicating a preference for a fully push approach (*n* = 23, 14.29%) or a more traditional entirely pull approach (*n* = 14, 8.70%).

#### JITAI protocol preferences

Approximately one-third of participants indicated a preference for three EMAs per day (*n* = 52, 32.30%), which was the number of EMAs delivered in this trial. A considerable number of participants (52.17%), however, endorsed a lower frequency EMA protocol: two EMAs (*n* = 48, 29.81%) or one EMA (*n* = 36, 22.36%). A smaller proportion of participants (15.53%) indicated they would prefer a higher frequency EMA protocol: four EMAs per day (*n* = 15, 9.32%) or five or more EMAs per day (*n* = 10, 6.21%). Almost a third of participants indicated a preference for a program length of one month (*n* = 45, 27.95%), which was the length of the current program. Approximately half of the sample (58.39%), however, indicated a preference for a longer program: 2 months (*n* = 22, 13.66%), 3–4 months (*n* = 26, 16.15%), 5–6 months (*n* = 19, 11.80%), and more than six months (*n* = 27, 16.77%). In contrast, only 13 participants (13.67%) indicated a preference for a Shorter program: 20 participants (12.42%) indicated a preference for a 2-week program duration and only 2 participants (1.24%) indicated a preference for a 1-week program duration.

#### Additional feedback and suggested improvements

Participants were invited to provide additional feedback or suggested improvements in an open-ended item at the end of the acceptability section in the post-intervention survey. Just under half of the participants who completed the post-intervention survey (*n* = 73 of 161; 45.34%) responded. Indicative responses are presented below.

##### General helpfulness

 Many participants indicated that they liked the app, were appreciative of the support it provided, and had no suggested improvements: “*It’s a great app. I wouldn’t change a lot on here. It’s helped me stop gambling so thank you.”* Many indicated that they found the app very helpful as it had helped them reduce or stop gambling:

I am absolutely happy with the benefits that I have received. Personally I don’t think any changes are necessary. For it (app) has changed my life (full reversal). Thank you for allowing me to participate….

##### Preferred app approach and JITAI preferences

Suggestions for improvements most often related to participants’ preferences for the approach of the app, the timing of the notifications, and the EMA content. Consistent with the earlier data, many again expressed a desire for a hybrid “pull and push” tailored approach in which they could complete an EMA and receive tailored intervention activities whenever they liked: “*I did want to be able to access all of the helpful tips at any time rather than having to wait for a check in.”* Sometimes, this was because they were unable to check in within the 2-hour window after the push notification arrived, sometimes because they were working or sleeping (for those on shiftwork): “*Mainly an option to go back into the check-in many times I was at work and couldn’t check in at that time. By the time I could check in it was too late”.* Other participants expressed a desire for a hybrid app so they could use the app at times they recognised they needed support:

I do see fit the idea of pull, because with the check ins, yes, the 3 times per day were sufficient on most, but for a recovering gambling addict like myself, the urge and desire to engage in gambling like activities can strike at any time (unfortunately). There were a few times where I wanted to check in, just to clear my head and come back to common ground, but the restrictions of only 3 check ins per day at set times halted this.

Conversely, a couple of participants indicated that the notifications or the reference to gambling in the EMAs reminded them of gambling when they were not vulnerable to gambling: “*I was thinking about gambling by checking in when I was busy with other tasks and not thinking about gambling.”*

Relatedly, a couple of participants expressed a desire for different notification timings: “*I went to bed at about 8pm when I need to work*,* however I missed some check ins because you send my notifications later so I would suggest looking at different timings”;* or to select their own check-in times: “*Would be helpful if you could set the times for the check ups. As I drive for work it was hard to answer when I got the questions. So if you could set the schedule for it that would be awesome.”* A couple of participants also wanted to have more than 2 h to complete the check-ins or a delay feature when they were unable to check in.

Several participants reported that the EMA items were repetitive, which for some resulted in reduced engagement: “*Overall it was a great approach to helping myself and fellow gamblers see the bigger picture. However*,* I think doing the same tasks and being given the same questions became repetitive and made me more reluctant to check in as often.”* A few participants therefore suggested asking different questions in each EMA or day, having the same items but with different wording, or having fewer questions. Several participants commented on the number of check-ins per day, all of whom indicated they were too frequent. Finally, a couple of participants failed to realise that the EMA items were employed to tailor the intervention, suggesting that they themselves did not offer insight or help.

##### Intervention content

Surprisingly few participants commented on the intervention content. For those who did, the suggestions for additions or improvement were extremely diverse. The only improvement that was suggested by more than one participant was the inclusion of additional urge management activities, such as games or distractions. However, several participants commented on the app delivering intervention content following the EMA via the intervention loop when they did not have the time or motivation, with a few acknowledging that they adjusted their answers to avoid the delivery of intervention content or close the intervention loop: “*I loved the app but it seemed that I had to complete the activities anytime I admitted to having gambling temptations and I didn’t always have the time or motivation for the activities so it made me adjust my answers.”*

##### Additional app features

 Many participants expressed a desire for additional features. The most common were behaviour change feedback, possibly in the form of graphs relating to gambling expenditure, time spent gambling, or potential savings and the provision of additional support from a personal coach, phone chat service, or person with lived experience, particularly at high-risk times. A couple of participants also mentioned the addition of other features, including an online discussion board, a journal or diary function, in-app rewards, and a bank of intervention activities so they could be directly accessed on demand.

##### Technical difficulties

Only a couple of participants reported technical difficulties, including not always receiving notifications, difficulty locating the home page, and the app not working after a phone update.

### Semi-structured interviews

Semi-structured interviews were conducted with a sub-sample of 11 participants to explore the acceptability of, and engagement in, the app in more depth. Thematic analysis of this data revealed two distinct themes: *The nature of an app such as GamblingLess: In-The-Moment to support reductions in gambling* and *Using GamblingLess: In-The-Moment to understand and change gambling behaviour.*

#### The nature of an app to support reductions in gambling

Participants discussed how and why *GamblingLess: In-The-Moment* worked to support someone wanting to reduce their gambling behaviour, including a feeling that someone was regularly *“checking in”* on them, and that support was “*always available”* on their phone, as well as discussions about how future iterations of the app could improve their experience, including “*tailoring to meet their personal needs”*, providing “*a seamless and holistic support experience”*, and supporting reductions in gambling that may be more suitable for *“early treatment seekers”* rather than those who had extensive treatment experience. These sub-themes are discussed below.

##### Checking in and always available

 While participants understood that daily check-ins were automated, and often commented on the repetitive nature of questions, they also found them comforting, as if a supportive friend was regularly checking in: “*Comforting thing to know that someone was checking in on my gambling.*” (GLO6: Female, 56, mild severity, high app use). Another key attraction for some was that support was always available through their smartphone: “*I always have my phone on me so having an app is definitely useful.*” (GLO5: Male, 38, moderate severity, moderate app use). Participants who gambled online further commented that it matched their main method of gambling, but that their phone was now supporting them to stop gambling.

##### Tailoring to meet personal needs

In terms of potential future iterations, participants discussed a variety of ways of tailoring or customising the app to meet their personal needs and preferences. As was articulated in the survey, participants strongly advocated for the ability to customise check-in notifications in terms of their timing and frequency. They talked about wanting to match their timing to suit their lifestyle and to target known times of vulnerability: “*I would’ve liked it to be like when I was gambling and like soon after I get paid.*” (GL11: Male, 45, severe severity, high app use). They also wanted to be able to check-in and access the intervention activities outside of the set check-in times, including at times of vulnerability or just when it was more convenient: “*When I’m having a bad moment*,* I would want the push notifications and when I wanted to just … go in and do a few activities to bring you back down to earth … would be good*.” (GLO6: Female, 56, mild severity, high app use). Although evaluating the acceptability of the trial conditions was not the focus of the interviews, one participant mentioned disappointment at receiving the no intervention control group message in the context of being able to check in and access intervention activities on-demand: *“I was really rapt with it. I was happy with the information and I would like to see though as I said*,* it was sometimes I really wanted to get into reading*,* but they said*,* “We’ll see you later.”* (GLO9: Male, 49, severe severity, high app use).

Participants wanted to be offered a range of intervention activities, but some expressed a desire to have control over their choice of activity at any given time and to be able to specify activities that they considered relevant to their circumstances: “*Instead of seeing 10 [activities]*,* I’ll just see the five that’s more relevant to me*” (GLO5: Male, 38, moderate severity, moderate app use). This included wanting to be able to specify activities based on their preferred forms or methods of gambling: “*There’s a bit of nuance involved with horse racing and different to pokies and general games of chance*,* so I feel like something specific on that might be helpful.*” (GLO3: Male, 23, severe severity, low app use). In contrast, other participants found the *Pick For Me* feature embedded within the app valuable as it directed them to activities they may not have chosen themselves:

“*It was something randomized and it more often than not showed me something that I didn’t know because if I did get something to choose*,* it would be something I wouldn’t be interested in and then that would sort of force me to choose something I was interested in. It would force me to choose something I wasn’t interested in and then I would know more about it.*” (GLO4: Male, 23, moderate severity, moderate app use).

The intervention loop had mixed feedback. Some participants could see clear benefits of this feature that encouraged continued engagement in activities until they were no longer vulnerable:

“*I had it [the intervention loop] a few times*,* um*,* and yeah sometimes I feel like I’ve gotten urges but it’s still asking me to do like another activity. I guess the looping is good you know once you finish one activity you if you still have an urge you definitely [should] do something else because you haven’t kind of cured the problem so …”* (GLO5: Male, 38, moderate severity, moderate app use).

This same participant went on to discuss how they managed the feature to suit their circumstances *“… if you’re using the app and it’s looping in*,* and if you think that*,* you know*,* ‘I don’t have that urge anymore’*,* I just turn the app off and go continue on with my life… it’s*,* you know*,* there’s no penalty.*” (GLO5: Male, 38, moderate severity, moderate app use). In contrast, negative reactions to this feature were more to do with not realising they could exit the loop when they wished (despite instructions to do so), than the feature itself:

“*It made you felt trapped inside of it*,* like*,* there was no salient exit*,* there was no clear exit just to go*,* okay*,* this is what I do*,* like I’ve done it. It didn’t give option. It felt like it was forcing me to do these things now because that didn’t work*,* I should try something else*.” (GLO2: Male, 40, moderate severity, high app use).

##### Providing a seamless and holistic support experience

 Thinking about future iterations, some participants were interested in the integration of additional app features to provide a seamless and holistic, wrap-around service. Again, suggestions included being able to connect to an e-coach, motivational messages, and the option to upload expenditure data from their online betting apps to avoid manual data entry. Another suggestion (which was also mentioned by one participant in the post-intervention open-ended item) was a feature that could issue an alert to users at points of vulnerability. For example, when they are walking into a gambling venue or are about to transfer money electronically to gamble: “*You want [an] alarm to go off when you walk in the door or something. Say ‘danger*,* danger’ … do you really want to go in there?*” (GLO7: Male, 69, severe severity, high app use).

Some participants also liked the idea of being able to connect to others via the app. Participants valued lived experience and said they would like the ability to connect to peers either through live chat or them uploading their personal stories: “*It would be really cool if you were able to access someone to talk to like that or if there was a community or people who were using it so you could see their posts and learn that way*.” (GLO3: Male, 23, severe severity, low app use). Participants also wanted to be able to link their app use to other help-seeking to streamline and service delivery: “*My whole experience of seeking help being into one place*.” (GLO2: Male, 40, moderate severity, high app use).

##### Suitability for early treatment-seekers

 Finally, a couple of participants with significant, long-term gambling issues suggested that the intervention activities in the app may be more appropriate for people who were newer to treatment. They each had a significant history of prior help-seeking and felt the content was familiar rather than new:

“*Yes*,* they were relevant but again probably a bit too general. But*,* in saying that*,* I think if it’s someone who’s seeking the first steps of something to help*,* then I think it’d be really good. It’s just I’ve been through a few programs*,* counselling*,* and stuff*,* that the concepts weren’t necessarily new to me I suppose*,* I would’ve wanted something more complex*.” (GLO3: Male, 23, severe severity, low app use).

#### Using the app to understand and change gambling behaviour

Almost all participants discussed the helpfulness of the app intervention in increasing their understanding of their gambling and, through this, the ability to change their gambling behaviour. This theme comprised four sub-themes: *A better awareness of gambling*,* Committing to new gambling goals*, *Learning new techniques*, and *Positive changes* since using the app.

##### A better awareness of gambling

Using the app led participants to have a better awareness of their gambling thoughts, behaviours, and habits. The app helped people to become more mindful in the moment and to consider the close relationship between their emotions and gambling: “*Great tool to bring consciousness back and all my awareness back to what I was doing*,* thinking*,* feeling*.” (GLO6: Female, 56, mild severity, high app use), including the ways emotions might drive gambling and how their gambling impacted their well-being: “*And you feel so guilty when you lose*,* and I just didn’t want to have that guilt anymore.*” (GLO8: Male, 74, severe severity, moderate app use). Participants discussed how the app facilitated self-reflection and a change in perspective regarding their gambling: “*Making me aware of my mindset when I did well*,* I was tempted to gamble.*” (GLO10: Female, 59, moderate severity, high app use). Recording past and current gambling behaviour was an important aspect of improved awareness, with participants having a new appreciation of how much they had been spending on gambling: “*I said because I’m not gambling. That’s a huge difference. If I had $2000 left over*,* I’d gamble that [in the past]. As I said*,* I haven’t done that.*” (GLO11: Male, 45, severe severity, high app use), as well as the value of money. Two participants also discussed becoming more aware of the impact their gambling was having on people close to them: “*It’s not just affecting me*,* definitely something I’m getting my head around.*” (GLO10: Female, 59, moderate severity, high app use).

##### Committing to new gambling goals

The 28-day trial was seen by some participants as long enough to change habits and commit to new gambling-related goals: “*28 days was kind of impactful…long enough to change some of my habits.*” (GLO5: Male, 38, moderate severity, moderate app use). Activities encouraged participants to examine their gambling behaviour and motivated commitment to new goals: “*The questions… they made me look at myself and say you know what*,* I can do this*.” (GLO11: Male, 45, severe severity, high app use). Interestingly, despite the three daily EMAs having a role in the tailoring rather than active treatment, participants commonly reported that they kept them accountable, forced them to be honest with themselves by reminding them of their personal goals and reinforcing these goals: “*It just checks in to see what you do in the morning*,* afternoon*,* and night. You got to be honest with it*,* you got to be honest with yourself.”* (GLO11: Male, 45, severe severity, high app use).

##### Learning new techniques

Participants reported that the app activities taught them many new techniques to help them control and reduce their gambling. Participants particularly recalled learning how to work through, manage and re-direct gambling urges: *“When I have an urge in my head*,* I’m thinking “it’s a wave*,* it’ll go away*,* let’s do something”*,* and this is all through the app teaching me that*.” (GLO9: Male, 49, severe severity, high app use).

People enjoyed using distraction activities to support them until urges passed: “*I do understand with the distraction activities*,* if you can distract yourself with five minutes*,* the urge just normally passes.*” (GLO11: Male, 45, severe severity, high app use). Interestingly, distraction activities could also be seen as an easy option compared to doing some of the other, more cognitively effortful activities:

“*I think when you*,* when you need help you want something that’s easy. You know*,* as you said*,* like [for some activities I need to] find a piece of paper and pen and think about what I wanted to write*,* it’s been hard*,* whereas*,* yeah*,* the apps take you somewhere and you just focus on this thing that the apps I guess recommend that you to do to forget about gambling that you know that that works and it’s easy and it takes five minutes and that anyone can do.”* (GLO5: Male, 38, moderate severity, moderate app use).

Participants also discussed how using the app had helped them to recognise and avoid high-risk situations: “*I stopped going to pubs because that’s where I’ll do it”* (GLO11: Male, 45, severe severity, high app use*).* One participant reported that working through the program helped him to the point that an old high-risk situation was no longer an issue: “*I can even just go near*,* within*,* the shopping centre near where some of the pokies are and it just doesn’t bother me anymore.’”* (GLO8: Male, 74, severe severity, moderate app use*).*

##### Positive changes

Importantly, participants talked about positive changes they had observed since using the app: “*I haven’t gambled in three weeks so far*,* so it could probably attribute with the app having some input in that*.” (GLO3: Male, 23, severe severity, low app use). Reduced gambling and increased feelings of control resulted in improved wellbeing, including improved mood: “*Yeah*,* I wake up in the morning and a lot of times I used to wake up and think I’ve got to go [to the pub]. Now*,* well*,* I’m happy…I don’t even think about it now.*” (GLO9: Male, 49, severe symptoms, high app use); financial freedom: “*I used to do payday loans and short-term loans … and now I’ve got none… which is absolutely a relief in itself.*” (GLO11: Male, 45, severe severity, high app use); and hope for the future “*There’s more hope now that I can save and go on holidays. I’ve got more hope for a future. I get the happy future.*” (GLO9: Male, 49, severe symptoms, high app use).

## Discussion

This study aimed to provide a comprehensive examination of the acceptability of, and engagement in, *GamblingLess: In-The-Moment* using app use and engagement indices, measures of app acceptability, and semi-structured interviews.

### App use and engagement

Consistent with previous research demonstrating that mHealth gambling interventions are generally acceptable [28, 44], an average of 36 eligible participants recruited monthly indicated that this JITAI was an attractive gambling support option. Participants had adequate exposure to intervention content during the 28-day MRT, spending over 30 min engaging and averaging nine intervention activities. However, app use declined over time and remained low during the six-month follow-up period, suggesting some disengagement. Overall, the evaluation demonstrated that the JITAI effectively tailored the delivery of the type, timing, and amount of support to individual needs.

### Type of support

Use of each intervention option varied in the MRT, with *Tackling Triggers* used most. Within each intervention option, participants tended to select activities based on their position on the intervention dashboard, which also occurred in the evaluation of the *GamblingLess* online program [32]. This suggests potential benefits from clearer descriptions of the activity content and/or randomisation of activities on each dashboard, which the JITAI platform could not support at the time. Despite this, participants found the intervention content supportive in increasing awareness and learning new techniques, indicating the type of support provided by the JITAI was well received.

### Timing of support

In *GamblingLess: In-The-Moment*, the timing of support is determined by three EMAs per day over 28 days. The EMA compliance rate was 31.72%, which is lower than the rates of 35–87% (average of 64%) in alcohol and substance use JITAIs [63] but consistent with other gambling JITAIs, including *GamblingLess*: *Curb Your Urge* (47% [33]) and *Gambling Habit Hacker* (30%; 31)). Although gambling EMA studies can achieve higher EMA compliance rates [52, 64], it remains unclear as to whether the relatively low EMA compliance rates observed in gambling JITAI studies are a result of the interventions or the populations they target. Regardless, these findings suggesting participants in this trial may not have been in a state of receptivity at the decision points. Receptivity, as defined in JITAI frameworks [17, 18], reflects willingness and ability to use the provided support. Given the lack of available evidence to inform the decision rules for *GamblingLess: In-The-Moment* [29, 35, 36], it remains unclear whether the timing and frequency of the EMAs target times of vulnerability [65].

This evaluation provided valuable insights into the preferred timing of EMAs (and subsequent support) among people wanting to quit or reduce their gambling. In the qualitative components of this study, many participants wanted the ability to customise EMA timing based on personal receptivity (e.g., late at night) and vulnerability (e.g., pay day) [29, 36]. Some suggested extending the 2-hour EMA completion window or adding a delay feature (beyond the existing *Snooze* feature) for more flexible EMA timing. Interestingly, Some participants completed multiple EMA outside the allowed 2-hour period (a platform limitation), indicating they engaged with the intervention on-demand. While customising EMA timing and on-demand access contrast with the assumption of “push” interventions that people are often unaware of their states of vulnerability or are unmotivated to access the requisite support [17, 18], the qualitative data demonstrated that some participants had good insight into when they needed support. Many preferred a hybrid push-pull approach, combining autonomous access and app-determined support, which would better address user needs while retaining flexibility.

The evaluation also revealed preferences for fewer daily EMAs and a longer program. While some participants found the EMA regularity comforting and supportive of accountability, the repetitiveness of EMAs may have decreased engagement. Future iterations of the app should consider alternative decision points or program lengths by taking client preferences into consideration and/or employing adaptive algorithms to identify appropriate decision points across time for each user [17, 18]. To reduce repetitiveness, participants suggested randomising, rewording, or changing the EMA items in each EMA or each day. Since only three tailoring are needed to determine intervention eligibility, future versions of the app offered under naturalistic conditions should include only these three items.

Adjusting the timing and frequency of EMAs to align with user states of receptivity could improve intervention engagement (motivation during treatment [66]) and reduce fatigue (cognitive or emotional weariness during the intervention [68]). Every attempt was made to enhance engagement via the delivery of individualised and evidence-based content in a non-judgemental communication style, as well as multiple intervention activities targeting the same cognitive process to minimise fatigue [20, 67–70]. However, the MARS engagement subscale received the lowest rating in usability testing, highlighting the need to further improve the entertainment, interest, interactivity and customisability of the app. These results align with previous research [34], suggesting mHealth gambling interventions could benefit from further studies on enhancing engagement and reducing fatigue.

#### Amount of support

In *GamblingLess: In-The-Moment*, the amount of the intervention content delivered was determined by the intervention loop, which was used by almost all participants. While many participants saw clear benefits of engaging with the app until they were no longer vulnerable, Some appeared to adjust their responses to exit the loop or close the app before reaching a score of 0. It remains unclear whether participants were satisfied with the amount of support they received, if the intervention eligibility threshold was too low, or if participants experienced intervention fatigue. Despite this, the findings suggest the intervention loop analysis, which demonstrated very large improvements in the secondary proximal outcomes, is relatively robust [36] as most participants did not reach a score of 0 to exit the loop.

### Subjective quality

Participants rated the subjective quality of *GamblingLess: In-The-Moment* over minimum acceptability requirements on the MARS. Although this score is slightly lower than its usability testing scores [35], it outperformed the free publicly available gambling support apps [20] and compared favourably to other gambling JITAIs [31, 34]. However, only one-third of participants indicated they would pay for the app, which is consistent with findings from previous gambling JITAI evaluations [31, 34]. This reluctance to pay for this gambling support app aligns with broader trends in health app usage [71, 72] and is particularly relevant for those financially impacted by gambling [20, 22]. Despite this, these positive subjective quality ratings are promising, particularly as they are positively associated with recovery or improvement in gambling symptom severity six months following treatment [36].

### Perceived impact

Participants also rated the perceived impact of *GamblingLess: In-The-Moment* highly, well surpassing minimum acceptability standards and outperforming free publicly available gambling support apps [20]. It also compares favourably to ratings for other JITAIs [30, 31, 34] and its own usability testing scores [35]. The app motivated participants to seek further help, which is consistent with the findings of some participants using the *Get More Support* quick link, indicating they were prepared to escalate the support they received, if needed [11]. Most importantly, most participants reported the app had decreased their gambling behaviour, which is reflected in the qualitative data and echoes the effectiveness of the app identified in the larger evaluation [36], although the suggestion it may be better suited for those in earlier stages of treatment requires further investigation.

### Implications for optimisation

Although *GamblingLess: In-The-Moment* demonstrated generally good acceptability across this evaluation, the findings highlight several avenues for its optimisation, with a view to enhancing user engagement and reducing fatigue. Low user engagement is arguably one of the biggest challenges for mental health and behaviour change apps [17, 18, 20, 73], with emerging research focusing on strategies to improve uptake and adherence [74–76]. Optimisation efforts should therefore prioritise enhancing engagement and minimising fatigue to improve treatment adherence, retention, and outcomes [19].

Participants preferred a hybrid model combining push (algorithm-initiated) and pull (participant-initiated) features. This approach would offer the benefit of regular notifications that are comforting but hold users accountable (push features) [17, 18] while allowing users to access support when they recognise vulnerability or are sufficiently motivated (pull features) [9, 15]. It could also help users with lower emotional self-awareness to enhance their recognition of these states over time [9–11, 15] and help them practice coping skills in real-life situations, thereby enhancing the generalisation of their learned skills to new settings [9–11, 15]. Incorporating “participant-determined features” may also promote autonomy through agency and control, and minimise disruption to users when they are unreceptive [18, 77]. Further research is required to determine the optimal way to balance planned support with personal choice [78].

Given the diversity in preferences for EMA frequency and timing and program length, adaptive algorithms using machine learning methods could be employed to continually re-adapt the decision points for each individual over time [12, 18, 79, 80]. Alternatively, user preferences could be incorporated, allowing users to adjust these settings as they progress through the treatment. Additionally, to reduce fatigue and improve engagement, only the three tailoring variables should be included to minimise the repetitiveness of EMA items.

Optimisation efforts targeting engagement and fatigue also have implications for the incorporation of proximal outcomes, tailoring variables, and intervention options [18, 19]. For example, an optimised version of this JITAI could incorporate engagement and fatigue markers as proximal outcomes and/or tailoring variables [19]. Adding support from lived experience peers, therapists, or e-coaches via chat, SMS or in-person could enhance engagement and reduce fatigue [13, 43, 81–83]. Specifically, guided mHealth interventions typically yield superior treatment outcomes [84] and the addition of peer-to-peer influence can facilitate supportive social interactions and increase feelings of relatedness and connectedness [10, 13, 43]. Qualitative feedback also suggested linking app use to other help-seeking services could streamline support and enable blended interventions, remote coaching (in which healthcare providers review data to facilitate the support they provide), and remote symptom monitoring (which can alert providers to concerning symptoms, inform them about the user’s condition, and improve the care they provide) [13]. However, since unguided interventions are the most cost-effective [43, 84] and participants were reluctant to pay for the app, further research is required to determine when additional support is valuable.

Participants also endorsed other additional features that have the potential to facilitate engagement [10, 12, 13, 18, 20, 43, 79, 81–83], including motivational messages, behaviour change feedback (e.g., gambling expenditure tracking), an online discussion board for peer support, a virtual computer coach, in-app rewards (e.g., achievement badges or points), and a journal or diary function. The review of free publicly available gambling support apps found that the number of features was not associated with MARS engagement scores, suggesting that the absolute number of features may be less important than the presence of specific features [20]. Incorporating even some of these features in future optimisation efforts could therefore serve to enhance app acceptability, engagement, and outcomes.

Finally, a feature is needed to allow users to exit the loop without adjusting their responses to the post-intervention EMA item. Alternatively, adjustments to the tailoring variable thresholds that determine intervention eligibility could be incorporated based on user preferences or adaptive tailoring. Additional features could include saving favourite activities and randomising the activities on each intervention dashboard.

These findings generally align with the directions for optimising *GamblingLess: In-The-Moment* from the MRT and within-group follow-up evaluation [36]. Over and above the implications for optimisation from the current study, these evaluations suggested that the *Tackling Triggers* and *Exploring Expectancies* options demonstrated efficacy and should be retained, but that there were mixed results for the *Curbing Cravings* option, suggesting the need for further evaluation of this module. Moreover, the findings from these evaluations suggested that individual differences, such as readiness to change, influenced outcomes, indicating that incorporating motivational strategies could enhance effectiveness.

### Strengths and limitations

This study offers the first comprehensive acceptability and engagement evaluation of one of the first gambling JITAIs using a mixed-methods approach examining app use and engagement indices, app acceptability measures, and semi-structured interviews. Based on a user-centred approach, the findings from end-users with lived experience will guide intervention optimisation to better meet their needs in the future [10, 43]. Such a comprehensive evaluation is important given the formative nature of gambling mHealth interventions [34, 38–40]. The large sample size, high post-intervention and follow-up evaluation rates suggest the findings are representative to the broader group of end-users, thereby enhancing their generalisability. The results highlight important implications for the next stages of research and future optimisation efforts.

The study has several limitations, including variability observed in the results, which may have resulted from the heterogeneity of treatment-seeking gambling populations in terms of demographic characteristics (e.g., gender, age), gambling chronicity, other substance use and mental health disorders such as depression and anxiety (e.g., [85]), suggesting future research is required to explore the factors associated enhanced user acceptability and engagement. EMA compliance rate was relatively low, which highlights the need for further research on intervention engagement and fatigue associated with this JITAI. Although initial usability testing demonstrated that all MARS subscales were rated well over the minimally acceptable standard [35], not all MARS subscales were included in this evaluation to reduce participant burden. Additionally, the study’s design did not directly align with contemporary acceptability frameworks such as the TFA [38]. Future trials should therefore include all MARS subscales, self-report measures of cognitive and affective engagement, as well as measures of TFA components.

Moreover, further evaluations are required to fully determine the acceptability of, and engagement in, the intervention in real-world settings because the extensive advertising, reimbursement, compliance protocols, microrandomisation, and convenience sampling employed in this trial may not reflect typical users and conditions. First, the advertising, reimbursement, and compliance protocols employed in this trial had the potential to enhance recruitment, engagement, and compliance. Second, while the aim of this study was to explore the acceptability of the JITAI, rather than the trial conditions, the evaluation was conducted in the context of the MRT in which participants were micro-randomised to a no intervention control condition 25% of the time, which has the potential to affect acceptability and engagement. Third, the convenience sampling resulted in predominantly EGM gamblers, which may limit the generalisability of the findings to participants reporting other types of gambling activities. However, the app was deliberately developed without reference to specific forms of gambling and similarly high EGM endorsement rates have been identified in a subsequent unpublished feasibility trial of this intervention under real-world conditions and previous Australian mHealth intervention trials (e.g., 31, 33, 86). Finally, although preliminary evidence suggests that adapting mHealth interventions for health promotion to different cultural populations may not yet be recommended [87], it may also be important for future research to explore the intervention’s cultural suitability and adaptation as its acceptability and effectiveness may vary significantly across different cultural or regional settings [88].

### Conclusion

This is one of the few comprehensive acceptability and engagement evaluations of a gambling mHealth intervention. The findings demonstrate that *GamblingLess: In-The-Moment* was an appealing and well-received option for gambling support, with participants generally perceiving it to be highly accessible and effective. The results are promising, especially compared to existing gambling support apps. However, they also underscore the iterative process involved in developing interventions within a JITAI framework and indicate potential ways to optimise, refine, and evaluate future iterations of the app, particularly in relation to enhancing user engagement and reducing fatigue. As the complexity of this JITAI increases with added contextual considerations or intervention tailoring options [79], machine learning models could be used to automatically adapt to new data in real time. This would allow for the continual re-adaptation of the decision rules for each user over time, thereby optimising the type, timing and amount of support provided [12, 16, 18, 19, 79, 80, 82].

## Supplementary Information

Supplementary Material 1

Supplementary Material 2

Supplementary Material 3

Supplementary Material 4

Supplementary Material 5

Supplementary Material 6

Supplementary Material 7

## Data Availability

Due to the sensitive nature of the study, data may be available on request to the corresponding author.

## Abbreviations

EMA: Ecological momentary assessment, a research method involving the collection of data regarding participant experiences in real-time, in the context in which these experiences naturally occur
JITAI: Just-in-time Adaptive interventions, interventions that use mobile and wearable devices to continuously monitor fluctuating internal states and social contexts to determine the type, amount, and timing of support required
MRT: Microrandomised trials, a form of sequential factorial design in which every participant serves as their own control
IRSAD: Index of relative socio-economic advantage and disadvantage, a measure of the socio-economic conditions of a given area with a standardised mean of 1000 to represent the national average, where higher scores reflect greater relative socio-economic advantage
TFA: Theoretical framework of acceptability, a multidimensional framework for appraising the acceptability of mHealth interventions, which comprises seven component constructs including affective attitude, burden, perceived effectiveness, ethicality, intervention coherence, opportunity costs, and self-efficacy
