# Supplementary material for: GamblingLess: In-The-Moment: a mixed-methods acceptability and engagement evaluation of a gambling just-in-time adaptive intervention
Source: Addict Sci Clin Pract. 2025 Oct 14;20:80. doi: 10.1186/s13722-025-00608-4 (PMC12522354; doi:10.1186/s13722-025-00608-4)
Supplement: Supplementary file 1 — Supplementary Material 1 [file 13722_2025_608_MOESM1_ESM.docx]

**Additional File 1**

| Table S1. Pre-Intervention Sample Characteristics | | | | | | | |
| --- | --- | --- | --- | --- | --- | --- | --- |
| Sample characteristic | | Mean | SD | Median | n | % | 95% CI |
| Age (years) | | 36.20 | 13.18 | 34.50 |  |  | (34.32, 38.07) |
| Gender | |  |  |  |  |  |  |
|  | Male |  |  |  | 127 | 66.15% | (59.12, 72.52) |
|  | Female |  |  |  | 64 | 33.33% | (26.99, 40.34) |
|  | Non-binary |  |  |  | 1 | 0.52% | (0.07, 3.65) |
| Ethnicity | |  |  |  |  |  |  |
|  | Australian |  |  |  | 145 | 75.52% | (68.90, 81.12) |
|  | Australian-mixed |  |  |  | 24 | 12.50% | (8.50, 18.01) |
|  | Other |  |  |  | 23 | 11.98% | (8.07, 17.42) |
| State of residence | |  |  |  |  |  |  |
|  | New South Wales |  |  |  | 94 | 48.96% | (41.91, 56.05) |
|  | Victoria |  |  |  | 51 | 26.56% | (20.76, 33.30) |
|  | Queensland |  |  |  | 24 | 12.50% | (8.50, 18.01) |
|  | South Australia |  |  |  | 15 | 7.81% | (4.75, 12.59) |
|  | Western Australia |  |  |  | 5 | 2.60% | (1.08, 6.14) |
|  | Australian Capital Territory |  |  |  | 2 | 1.04% | (0.26, 4.10) |
|  | Tasmania |  |  |  | 1 | 0.52% | (0.07, 3.65) |
| Annual personal gross income (AUD$) | |  |  |  |  |  |  |
|  | Less than $25,000 |  |  |  | 24 | 12.50% | (8.50, 18.01) |
|  | $25,000 to $49,999 |  |  |  | 31 | 16.15% | (11.56, 22.09) |
|  | $50,000 to $74,999 |  |  |  | 51 | 26.56% | (20.76, 33.30) |
|  | $75,000 to $99,999 |  |  |  | 38 | 19.79% | (14.72, 26.07) |
|  | $100,000 to $124,999 |  |  |  | 22 | 11.46% | (7.64, 16.83) |
|  | $125,000 to $149,999 |  |  |  | 16 | 8.33% | (5.15, 13.21) |
|  | $150,000 to $174,999 |  |  |  | 6 | 3.13% | (1.40, 6.81) |
|  | $175,000 to $199,999 |  |  |  | 3 | 1.56% | (0.50, 4.76) |
|  | $200,000 or more |  |  |  | 1 | 0.52% | (0.07, 3.65) |
| SEIFA (IRSAD)^a^ | | 1011.10 | 75.93 | 1008.00 |  |  | (1000.26, 1021.94) |
| Problem gambling activity^b^ | |  |  |  |  |  |  |
|  | Electronic gaming machines |  |  |  | 126 | 65.63% | (58.59, 72.04) |
|  | Racing |  |  |  | 103 | 53.65% | (46.52, 60.62) |
|  | Sports or events |  |  |  | 91 | 47.40% | (40.39, 54.51) |
|  | Number games |  |  |  | 40 | 20.83% | (15.64, 27.20) |
|  | Table games |  |  |  | 37 | 19.27% | (14.27, 25.51) |
|  | Informal private games |  |  |  | 17 | 8.85% | (5.56, 13.82) |
| G-SAS Gambling symptom severity | | 30.64 | 8.26 | 31.50 |  |  | (29.47, 31.82) |
| TLFB Past-month gambling behaviour | |  |  |  |  |  |  |
|  | Frequency | 8.05 | 5.66 | 7.00 |  |  | (7.25, 8.86) |
|  | Expenditure ($AUD) | 3313.50 | 4258.00 | 2150.00 |  |  | (2707.37, 3919.63) |
| SEIFA: Socio-Economic Indexes for Areas; IRSAD=Index of Relative Socio-Economic Advantage and Disadvantage; G-SAS: Gambling Symptom Assessment Scale; TLFB: TimeLine Follow-Back  ^b^Multiple response options allowed.  n=192 (pre-intervention analytic sample) | | | | | | | |
