# Supplementary material for: GamblingLess: In-The-Moment: a mixed-methods acceptability and engagement evaluation of a gambling just-in-time adaptive intervention
Source: Addict Sci Clin Pract. 2025 Oct 14;20:80. doi: 10.1186/s13722-025-00608-4 (PMC12522354; doi:10.1186/s13722-025-00608-4)
Supplement: Supplementary file 2 — Supplementary Material 2 [file 13722_2025_608_MOESM2_ESM.docx]

**Additional File 2**

| Table S2. Sample Characteristics for the Semi-Structured Interviews | | | | | | |
| --- | --- | --- | --- | --- | --- | --- |
| Participant ID number | Gender | Age | State of residence | App use | G-SAS gambling symptom severity | Problem gambling activity |
| GL01 | Female | 62 | NSW | Low | Severe | Number games, EGMs |
| GL02 | Male | 40 | VIC | High | Moderate | Number games, racing, sports |
| GL03 | Male | 23 | NSW | Low | Severe | Racing, sports |
| GL04 | Male | 23 | VIC | Moderate | Moderate | Racing, sports |
| GL05 | Male | 38 | VIC | Moderate | Moderate | Racing, sports |
| GL06 | Female | 56 | NSW | High | Mild | Number games, EGMs, racing, sports |
| GL07 | Male | 69 | VIC | Low | Severe | EGMs |
| GL08 | Male | 74 | QLD | Moderate | Severe | EGMs |
| GL09 | Male | 49 | NSW | High | Severe | EGMs |
| GL10 | Female | 59 | SA | High | Moderate | Number games, EGMs |
| GL11 | Male | 45 | VIC | High | Severe | Table games, racing, sports |
| n=11 comprising the subsample of participants completing the semi-structured interviews. App use was classified as low (4-8 EMAs completed), moderate (9-58 EMAs completed), or high (59+ EMAs completed). | | | | | | |
