# Supplementary material for: GamblingLess: In-The-Moment: a mixed-methods acceptability and engagement evaluation of a gambling just-in-time adaptive intervention
Source: Addict Sci Clin Pract. 2025 Oct 14;20:80. doi: 10.1186/s13722-025-00608-4 (PMC12522354; doi:10.1186/s13722-025-00608-4)
Supplement: Supplementary file 3 — Supplementary Material 3 [file 13722_2025_608_MOESM3_ESM.docx]

**Additional File 3**

**Semi-Structured Interview Schedule**

**Welcome:** Thank you for making time to talk to me today. I am looking forward to hearing about experiences with the GamblingLess: In-The-Moment App. Your experiences will help us to understand the strengths of the app and where we can make improvements for other people who want to reduce their gambling.

**Verbal consent**: Before we begin, we need to obtain your explicit verbal consent to participate in this interview. I will read aloud some statements and then obtain your verbal consent.

Firstly, do you have any questions regarding the plain language statement that was provided to you by email and when you responded to the invitation to take part in the interview?

This is interview with participant number ____ _____ [from tracker]

On __ ________

By agreeing to participate this interview, you are declaring that:

- You have read and understood the Participant Information Sheet
- You have been given a copy of the Plain Language Statement and Consent Form to keep (by email).
- You freely agree to participate in this project according to the conditions in the Participant Information Sheet.
- This involves taking part in an audio-recorded interview with a trained research fellow talking about your experience using the **GamblingLess: In-The-Moment** app over the 4-week trial period. You do not need to discuss anything you are not comfortable with talking about and can stop at any time.
- You understand that the research team has agreed not to reveal your identity and personal details, including where information about this project is published, or presented in any public form.

**Interview outline:** Your interview will be around 30-45 minutes and we will cover 3 main areas. First I’ll ask some questions about your first experiences using the app then I’m going to ask some questions about the activities and features in the app. I will finish with some general questions about personal changes having used the app and your overall impressions of the app.

As I mentioned in the consent you only need to talk about things you are comfortable with and you can stop at any time.

**The first set of questions relate to your first experiences with GamblingLess: In-The-Moment and when you first downloaded and used the app**.  

1. **When you first went to the app store/downloaded the app do you remember what it was you were searching for?**

- Prompt – what were your goals at that time? What did you want to change/get help for?
- Prompt - What made you select GamblingLess: In-The-Moment ? (app modality, what it is trying to do, credibility)
- Prompt – what made you think GamblingLess: In-The-Moment would meet your goals?

1. **I want to now turn to the check-in process.** You might remember that three times a day you received notifications on your phone to check in. And we sent these for 28 days. What was that like for you?
   - Prompt (number, timing) – were there too few, too many, were they the right questions for you? Were the timings ok across the day?
   - Prompt (trial duration) – Was getting notifications for 28 days too long, too short, or about right?
   - Prompt (motivators and barriers to checking in) - Did you always respond to the notifications? If no/yes why...
     - What made you respond?
     - Was there a day or few days that you stopped checking in?
     - If stopped at some point why -What was happening in your life / gambling when you stopped checking in?)
     - If you went a few days without checking in what made you come back again?
   - Prompt (event record, tracking gambling) - What about entering the gambling information each time (did you gamble, how much?)
     - Did you ever forget? What did you do then?
     - Would you prefer to have entered it straight away when you gambled or was later ok?
     - Can you think of any other ways that you would have liked to be able to track your gambling spending in the app?

3. **Did the check-in link you into an appropriate interactive activity that helped you in the moment?**

- Probe: Were there times when you thought the activity did not make sense for your situation?

**Now I’d like to talk a bit about the activities in the app itself.**

***Intervention activities***

1. **How helpful did you find the interactive activities in the app?**

- Prompt – Were any specific activities that you found really helpful? Or found not very helpful? Were they easy to do? Hard or painful to do?
- Prompt - What specific techniques, tips or suggestions have you gotten from the app? (if any).
  - Do you think you’ll continue to use these in the future when you have an urge, etc.?
- Prompt – Are there any other activities that you’d like to see included in the app?

***Specific app features***

1. **What was your overall experience of using the app in terms of using the interactive activities?**

- Prompt – intervention loop? Did you like that idea? What made you bail out?
- Prompt – Did you Know? (Delivers psyched)
- Prompt – Pick for Me? Random selection of tailored activities?
- Prompt – Get more support? (lots of places offered option to access more support) and if applicable use following prompts.
  - Did it prompt you to think about getting more support? Did you act?
  - If accessed other support while using the app - How did they find going to counselling and using the app at the same time?

1. **Were there any other features that you’d like to see included in the app?**

(if needed prompt for some of the list below)

- Being able to access the program any time you choose?
- in-person support/guidance/encouragement (e.g., therapists, lived experience, e-coach to provide additional support, information, feedback, tech support, explain things, reminders, motivation)? Via what modality (e.g., SMS, chat, email, telephone, video-conferencing, in person)?
- Online discussion board (e.g., offer and receive support from other people)?
- Receiving additional messages to help you stay motivated?
- Having access to a virtual computer coach (AI) that you can talk to?
- Getting feedback about changes to your gambling, such as graphs?
- Getting in-app rewards, like achievement badges or points?
- Being able to save your favourite activities?

1. **Apps can be ‘pull’ (access the content any time you want), ‘push’ (app assesses you and decides whether you need to access the content), or both.** The GamblingLess: In-The-Moment app was a ‘push’ app in the first 28 days, but will be ‘pull’ over the next six months. (if needed, prompt for some of the list below). Would you prefer that the app was set up so that you:

- Have all activities available for you any time you want (e.g., like Reset; most apps)
- Complete a check-in any time you want and get a tailored activity (user-initiated check-ins; next 6 months)
- Complete a check-in when you get a notification (28 MRT)
- Some combination of the above

***Generic helpfulness***

**You’ve given us lots of information today. Just a last couple of questions. Thinking overall about the whole app:**

1. **What changes have you noticed in your gambling or other parts of your life** **as a result of using the app**? (e.g., if needed - gambling less often/less $; increased wellbeing, less conflict – depending on change).
   - Prompt: Has anything changed for the worse?
   - Prompt: Is there anything that you wanted to change that hasn’t since you started using the app?
2. **What about the look and feel of the app?** Can you say something about your overall impressions? Did you like the images, colours, cartoon people, interactivity, videos?
3. **What else would you like to tell us about your experiences of the app?**
4. **Would you like to review the transcript of this interview?**
