# Supplementary material for: GamblingLess: In-The-Moment: a mixed-methods acceptability and engagement evaluation of a gambling just-in-time adaptive intervention
Source: Addict Sci Clin Pract. 2025 Oct 14;20:80. doi: 10.1186/s13722-025-00608-4 (PMC12522354; doi:10.1186/s13722-025-00608-4)
Supplement: Supplementary file 4 — Supplementary Material 4 [file 13722_2025_608_MOESM4_ESM.docx]

**Additional File 4**

| Table S4. Timing of App Use | | | | | | |
| --- | --- | --- | --- | --- | --- | --- |
|  | | Mean | SD | Median | IQR 25% | IQR 75% |
| Time of day | |  |  |  |  |  |
|  | Morning | 8.56 | 9.16 | 4 | 1 | 17 |
|  | Afternoon | 8.99 | 9.74 | 4 | 1 | 18 |
|  | Evening | 9.09 | 9.70 | 3.5 | 1 | 19.5 |
| Week of MRT period | |  |  |  |  |  |
|  | Week 1 | 8.99 | 6.82 | 7 | 2 | 16 |
|  | Week 2 | 6.28 | 7.39 | 2 | 0 | 13 |
|  | Week 3 | 5.72 | 7.38 | 0 | 0 | 12 |
|  | Week 4 | 5.65 | 7.61 | 0 | 0 | 13 |
| n=192 (analytic sample) | | | | | | |
