# Supplementary material for: GamblingLess: In-The-Moment: a mixed-methods acceptability and engagement evaluation of a gambling just-in-time adaptive intervention
Source: Addict Sci Clin Pract. 2025 Oct 14;20:80. doi: 10.1186/s13722-025-00608-4 (PMC12522354; doi:10.1186/s13722-025-00608-4)
Supplement: Supplementary file 5 — Supplementary Material 5 [file 13722_2025_608_MOESM5_ESM.docx]

**Additional File 5**

| Table S5. Number of Intervention Activities Used | | | | | |  |  |  |  |
| --- | --- | --- | --- | --- | --- | --- | --- | --- | --- |
|  | Mean | SD | Median | IQR 25% | IQR 75% | Min | Max | Total | % participants |
| Overall | 9.11 | 13.23 | 6 | 2 | 11 | 1 | 128 | 1503 | - |
| Intervention option 1: Curbing Cravings | 3.67 | 3.81 | 2 | 1 | 5 | 1 | 22 | 327 | 82.41 |
| Intervention option 2: Tackling Triggers | 5.18 | 9.38 | 3 | 2 | 6 | 1 | 102 | 668 | 93.48 |
| Intervention option 3: Exploring Expectancies | 4.34 | 5.49 | 2 | 1 | 5 | 1 | 33 | 508 | 90.00 |
| n=192 (analytic sample) | | | | | | | | | |
