# Supplementary material for: GamblingLess: In-The-Moment: a mixed-methods acceptability and engagement evaluation of a gambling just-in-time adaptive intervention
Source: Addict Sci Clin Pract. 2025 Oct 14;20:80. doi: 10.1186/s13722-025-00608-4 (PMC12522354; doi:10.1186/s13722-025-00608-4)
Supplement: Supplementary file 6 — Supplementary Material 6 [file 13722_2025_608_MOESM6_ESM.docx]

**Additional File 6**

| Table S6. Intervention Activities Selected | |  |  |  |  |  |  |  |  |  |  |
| --- | --- | --- | --- | --- | --- | --- | --- | --- | --- | --- | --- |
|  | | Mean | SD | Median | IQR 25% | IQR 75% | Min | Max | Total | % participants | % EMAs |
| Intervention option 1: Curbing Cravings | |  |  |  |  |  |  |  |  |  |  |
| 1. | Delay and distract | 1.69 | 1.56 | 1 | 1 | 2 | 1 | 11 | 81 | 44.44 | 22.96 |
| 2. | Breathe through it | 1.90 | 1.97 | 1 | 1 | 2 | 1 | 8 | 55 | 26.85 | 16.31 |
| 3. | Tense and relax | 1.33 | 0.62 | 1 | 1 | 2 | 1 | 3 | 20 | 13.89 | 6.04 |
| 4. | Anti-autopilot | 1.29 | 0.74 | 1 | 1 | 1 | 1 | 4 | 40 | 28.70 | 12.08 |
| 5. | Urge surfing | 1.33 | 0.62 | 1 | 1 | 2 | 1 | 3 | 20 | 13.89 | 5.74 |
| 6. | Fast-forward | 1.31 | 0.68 | 1 | 1 | 1 | 1 | 4 | 34 | 24.07 | 9.97 |
| 7. | Thinking traps | 1.50 | 0.71 | 1.5 | 1 | 2 | 1 | 2 | 3 | 1.85 | 0.91 |
| 8. | Pros and cons | 1.25 | 0.77 | 1 | 1 | 1 | 1 | 4 | 20 | 14.81 | 6.04 |
| 9. | The benefits | 1.43 | 0.94 | 1 | 1 | 1 | 1 | 4 | 20 | 12.96 | 6.04 |
| 10. | Ten steps to stay safe | 1.17 | 0.38 | 1 | 1 | 1 | 1 | 2 | 34 | 26.85 | 9.67 |
| Intervention option 2: Tackling Triggers | |  |  |  |  |  |  |  |  |  |  |
| Financial pressures | |  |  |  |  |  |  |  |  |  |  |
| 1. | Money check | 1.48 | 0.86 | 1 | 1 | 2 | 1 | 5 | 74 | 36.23 | 12.68 |
| 2. | Financial benefits | 1.20 | 0.41 | 1 | 1 | 1 | 1 | 2 | 24 | 14.49 | 4.04 |
| 3. | Debate captain | 1.08 | 0.28 | 1 | 1 | 1 | 1 | 2 | 27 | 18.12 | 4.78 |
| 4. | Financial goals | 1.04 | 0.21 | 1 | 1 | 1 | 1 | 2 | 24 | 16.67 | 4.41 |
| 5. | Fast forward | 1.05 | 0.22 | 1 | 1 | 1 | 1 | 2 | 21 | 14.49 | 3.86 |
| Unpleasant emotions | |  |  |  |  |  |  |  |  |  |  |
| 1. | Defusing difficult thoughts | 1.48 | 1.22 | 1 | 1 | 2 | 1 | 7 | 40 | 19.57 | 7.35 |
| 2. | Enjoyable activities | 1.40 | 0.82 | 1 | 1 | 1.5 | 1 | 4 | 28 | 14.49 | 4.96 |
| 3. | Living in the moment | 1.38 | 0.67 | 1 | 1 | 2 | 1 | 3 | 29 | 15.22 | 4.96 |
| 4. | Tense and relax | 1.36 | 1.34 | 1 | 1 | 1 | 1 | 6 | 19 | 10.14 | 3.49 |
| 5. | Pros and cons | 1.42 | 0.79 | 1 | 1 | 1.5 | 1 | 3 | 17 | 8.70 | 2.76 |
| Social pressure to gamble | |  |  |  |  |  |  |  |  |  |  |
| 1. | The “No” word | 1.59 | 1.70 | 1 | 1 | 1 | 1 | 8 | 27 | 12.32 | 4.41 |
| 2. | Just say no | 1.59 | 1.37 | 1 | 1 | 1 | 1 | 6 | 27 | 12.32 | 4.78 |
| 3. | Do’s and Don’ts | 2.13 | 3.18 | 1 | 1 | 1 | 1 | 10 | 17 | 5.80 | 2.57 |
| 4. | Planning ahead | 2.25 | 2.43 | 1 | 1 | 2.5 | 1 | 8 | 18 | 5.80 | 2.94 |
| 5. | Safe and dangerous contacts | 2.00 | 1.73 | 1 | 1 | 3 | 1 | 6 | 18 | 6.52 | 3.31 |
| Testing control | |  |  |  |  |  |  |  |  |  |  |
| 1. | Willpower breakdown | 1.38 | 0.93 | 1 | 1 | 2 | 1 | 7 | 76 | 39.86 | 12.87 |
| 2. | Debate captain | 1.13 | 0.46 | 1 | 1 | 1 | 1 | 3 | 26 | 16.67 | 4.60 |
| 3. | Seemingly irrelevant decisions | 1.24 | 0.52 | 1 | 1 | 1 | 1 | 3 | 31 | 18.12 | 5.51 |
| 4. | The slip chain | 1.15 | 0.37 | 1 | 1 | 1 | 1 | 2 | 30 | 18.84 | 5.51 |
| 5. | Safe gambling guidelines | 1.56 | 1.20 | 1 | 1 | 2 | 1 | 6 | 28 | 13.04 | 4.23 |
| Conflict with others | |  |  |  |  |  |  |  |  |  |  |
| 1. | My style | 1.36 | 0.73 | 1 | 1 | 2 | 1 | 4 | 30 | 15.94 | 5.51 |
| 2. | Choice spinner | 1.36 | 0.92 | 1 | 1 | 1 | 1 | 4 | 15 | 7.97 | 2.39 |
| 3. | Mix ‘n match | 1.67 | 1.15 | 1 | 1 | 3 | 1 | 3 | 5 | 2.17 | 0.92 |
| 4. | “I” statements | 1.11 | 0.33 | 1 | 1 | 1 | 1 | 2 | 10 | 6.52 | 1.65 |
| 5. | Do’s and don’ts | 1.40 | 0.55 | 1 | 1 | 2 | 1 | 2 | 7 | 3.62 | 1.29 |
| Intervention option 3: Exploring expectancies | |  |  |  |  |  |  |  |  |  |  |
| Excitement expectancies | |  |  |  |  |  |  |  |  |  |  |
| 1. | Feedback | 1.36 | 0.78 | 1 | 1 | 1 | 1 | 4 | 38 | 21.54 | 7.16 |
| 2. | Tense and relax | 1.54 | 1.10 | 1 | 1 | 1.5 | 1 | 5 | 37 | 18.46 | 6.96 |
| 3. | Pros and cons | 1.27 | 0.55 | 1 | 1 | 1 | 1 | 3 | 28 | 16.92 | 5.22 |
| 4. | Exciting activities | 1.30 | 0.88 | 1 | 1 | 1 | 1 | 5 | 30 | 17.69 | 5.61 |
| 5. | Fast forward | 1.13 | 0.35 | 1 | 1 | 1 | 1 | 2 | 17 | 11.54 | 3.29 |
| 6. | Anti-autopilot | 1.22 | 0.44 | 1 | 1 | 1 | 1 | 2 | 11 | 6.92 | 2.13 |
| Escape expectancies | |  |  |  |  |  |  |  |  |  |  |
| 1. | Feedback | 1.50 | 0.96 | 1 | 1 | 2 | 1 | 4 | 33 | 16.92 | 5.22 |
| 2. | Tense and relax | 1.62 | 1.19 | 1 | 1 | 2 | 1 | 5 | 21 | 10.00 | 3.87 |
| 3. | Pros and cons | 1.72 | 1.71 | 1 | 1 | 2 | 1 | 8 | 31 | 13.85 | 5.80 |
| 4. | Enjoyable activities | 1.38 | 1.02 | 1 | 1 | 1 | 1 | 5 | 22 | 12.31 | 4.26 |
| 5. | Defusing difficult thoughts | 1.36 | 1.08 | 1 | 1 | 1 | 1 | 5 | 19 | 10.77 | 3.68 |
| 6. | Living in the moment | 1.21 | 0.58 | 1 | 1 | 1 | 1 | 3 | 17 | 10.77 | 3.09 |
| Money expectancies | |  |  |  |  |  |  |  |  |  |  |
| 1. | Feedback | 1.50 | 1.00 | 1 | 1 | 2 | 1 | 5 | 42 | 21.54 | 7.93 |
| 2. | Money check | 1.80 | 2.29 | 1 | 1 | 2 | 1 | 15 | 74 | 31.54 | 14.12 |
| 3. | The chasing thinking trap | 1.19 | 0.49 | 1 | 1 | 1 | 1 | 3 | 31 | 20.00 | 5.80 |
| 4. | Debate captain | 1.20 | 0.41 | 1 | 1 | 1 | 1 | 2 | 18 | 11.54 | 3.48 |
| 5. | Fast forward | 1.11 | 0.32 | 1 | 1 | 1 | 1 | 2 | 20 | 13.85 | 3.87 |
| 6. | Anti-autopilot | 2.88 | 2.46 | 2 | 1 | 4 | 1 | 12 | 161 | 43.08 | 3.68 |
| n=192 (analytic sample) | | | | | | | | | | | |
