# Supplementary material for: GamblingLess: In-The-Moment: a mixed-methods acceptability and engagement evaluation of a gambling just-in-time adaptive intervention
Source: Addict Sci Clin Pract. 2025 Oct 14;20:80. doi: 10.1186/s13722-025-00608-4 (PMC12522354; doi:10.1186/s13722-025-00608-4)
Supplement: Supplementary file 7 — Supplementary Material 7 [file 13722_2025_608_MOESM7_ESM.docx]

**Additional File 7**

| Table S7. Intervention Loop Use | | | | | | | | |
| --- | --- | --- | --- | --- | --- | --- | --- | --- |
|  | | Mean | SD | Median | IQR 25% | IQR 75% | Min | Max |
| Intervention option 1: Curbing Cravings (n=108) | |  |  |  |  |  |  |  |
|  | Times used loop | 2.34 | 3.05 | 1 | 1 | 3 | 0 | 16 |
|  | Times left before reaching score of 0 | 2.33 | 3.14 | 1 | 1 | 3 | 0 | 16 |
| Intervention option 2: Tackling Triggers (n=138) | |  |  |  |  |  |  |  |
|  | Times used loop | 3.17 | 3.88 | 2 | 1 | 4 | 0 | 29 |
|  | Times left before reaching score of 0 | 2.87 | 3.87 | 2 | 1 | 3 | 0 | 29 |
| Intervention option 3: Exploring Expectancies (n=130) | |  |  |  |  |  |  |  |
|  | Times used loop | 3.00 | 4.10 | 1.5 | 1 | 3 | 0 | 26 |
|  | Times left before reaching score of 0 | 2.68 | 3.98 | 1 | 1 | 3 | 0 | 26 |
| n=192 (analytic sample) | | | | | | | | |
